# CRISPR-mediated targeting of the *LMNA* c.745C>T mutation enhances survival and cardiac function in congenital muscular dystrophy

**DOI:** 10.1016/j.omta.2025.201653

**Published:** 2025-12-26

**Authors:** Déborah Gómez-Domínguez, Carolina Epifano, Iván Hernández, Borja Vilaplana-Martí, Alberto Martín, Sandra Amarilla-Quintana, Sergi Cesar, Antonio de Molina-Iracheta, Miguel Sena-Esteves, Georgia Sarquella-Brugada, Ignacio Pérez de Castro

**Affiliations:** 1 Instituto de Investigación de Enfermedades Raras, Instituto de Salud Carlos III, Ctra. Majadahonda-Pozuelo km2.2, 28029 Madrid, Spain; 2 Fundación Andrés Marcio, niños contra la Laminopatía, C/Núñez de Balboa, 11, 28001 Madrid, Spain; 3 Pediatric Arrhythmias, Inherited Cardiac Diseases and Sudden Death Unit, Hospital Sant Joan de Deu, 08950 Barcelona, Spain; 4 Medicina Comparada, Centro Nacional de Investigaciones Cardiovasculares, 28029 Madrid, Spain; 5 UMass Chan Medical School, Department of Neurology and Horae Gene Therapy Center, Worcester, MA 01605, USA

**Keywords:** *LMNA*, L-CMD, laminopathies, CRISPR-Cas9, gene therapy, rare disease

## Abstract

*LMNA*-associated congenital muscular dystrophy is a currently incurable rare genetic disorder characterized by early-onset muscle weakness, dilated cardiomyopathy, and respiratory failure, resulting from mutations in the *LMNA* gene. In this study, we assessed the potential of a CRISPR-mediated strategy to eliminate the mutant allele *Lmna* c.745C>T, p.R249W using a mutation-specific guide (sg745T). Results from R249W-mutation-carrying cellular models showed specific activity of the Cas9/sg745T complex toward the mutant allele. This property varied depending on the concentration of CRISPR components, with a loss of specificity observed with increased dosage. We tested this strategy *in vivo* using adeno-associated virus delivery in *Lmna*^*R249W*^ mice. Despite being associated with a modest CRISPR activity, this therapeutic approach led to a 10% (non-significant) increase in the survival of R249W homozygous mice. Interestingly, a comparable CRISPR activity significantly ameliorated the cardiac pathology observed in *Lmna*^*+/R249W*^ animals, resulting in a significant 24.3% extension of their median survival. These results represent the first therapeutic validation of a CRISPR-Cas9-mediated gene editing strategy for the treatment of *LMNA*-associated congenital muscular dystrophy.

## Graphical abstract

**Figure:**
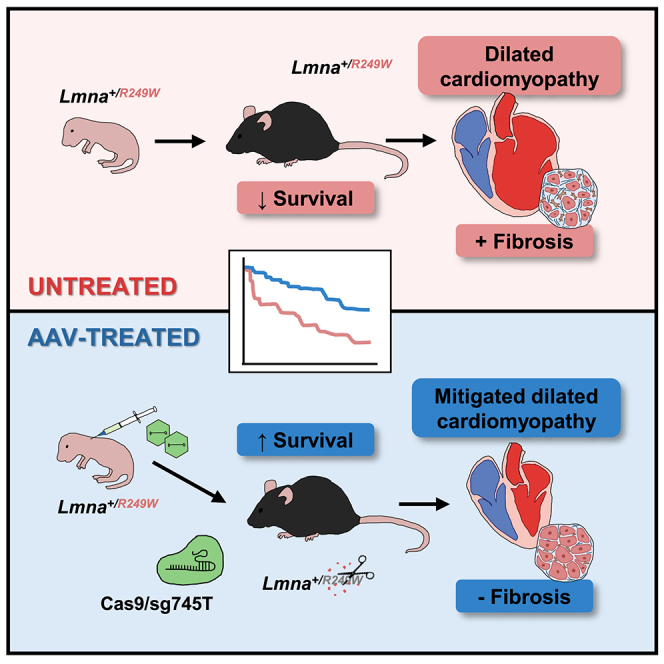

## Teaser

Pérez de Castro and colleagues validate a CRISPR-Cas9-based therapeutic strategy to target the *Lmna* c.745C>T mutant allele in cellular models and mice, improving survival and cardiac pathology in *LMNA*-associated congenital muscular dystrophy.

## Introduction

*LMNA*-related congenital muscular dystrophy (L-CMD) is an autosomal-dominant myopathy inherited genetically, with an extremely rare overall prevalence of less than 1 in 1,000,000 (ORPHA:157973). L-CMD patients display severe clinical manifestations of skeletal muscle laminopathies, characterized by an early onset and a rapid progression.^1^ The disease is characterized by motor development delay due to significant skeletal muscle weakness observed in the first months of life or even during the fetal period. Symptoms vary in severity and include elevated creatine kinase levels,^2^ dropped head syndrome,^3^ joint contractures,^4^ and cardiac and respiratory complications over time that lead to sudden death.^5^

L-CMD is one of the 15 rare diseases designated as laminopathies, which are associated with abnormalities in the nuclear lamina and are caused by mutations in the main components of this subcellular structure.^6^ Most L-CMD patients have mutations located in exons 1, 4, 6, and 7 of the *LMNA* gene, which coincide with the coils of the head and central domains of the protein and the immunoglobulin domain of the tail.^5^^,^^7^ The most frequent variant associated with L-CMD is the c.745C>T missense mutation, located in exon 4, that results in an arginine to tryptophan substitution at position 249 (p.R249W).^5^^,^^8^^,^^9^ To study L-CMD underlying mechanisms and test therapeutic strategies, we have developed and characterized an *Lmna*^*R249W*^ mouse model (unpublished data). *Lmna*^*R249W/R249W*^ mice show severe growth delay leading to premature death at around 50 days of age. On the other hand, *Lmna*^*+/R249W*^ mice recapitulate the cardiac abnormalities developed by L-CMD patients, their genetic equivalents, consisting of progressive dilated cardiomyopathy with left ventricular dilation and reduced cardiac function leading to sudden death.

Currently, there is no cure for L-CMD, and treatment focuses on symptom management. Palliative approaches include exercise programs, mechanical aids, surgical intervention, and monitoring of cardiac and respiratory functions.^10^^,^^11^ The disease, despite improvements in life expectancy, remains incurable, emphasizing the need for the development of effective therapies. Several pre-clinical studies have assessed the therapeutic potential of different strategies to treat laminopathies. Small molecule drugs targeting the MAPK and mTOR pathways as well as NAT10 have been tested for treatment of laminopathies.^12^^,^^13^^,^^14^ The p38α inhibitor PF-07265803 (previously known as ARRY-797) is the only drug that progressed to the clinical trial stage for the treatment of *LMNA*-related dilated cardiomyopathy (ClinicalTrial.gov NCT03439514). Unfortunately, this program has been recently discontinued because interim results indicated it would not meet the primary endpoint. Gene therapies have also been explored to treat some laminopathies being the main focus on Hutchinson-Gilford progeria syndrome (HGPS). Promising results have been obtained for this LMNA-related disease using CRISPR 1.0 technology^15^^,^^16^ and base editors,^17^^,^^18^^,^^19^^,^^20^^,^^21^ a more advanced CRISPR-based approach, in different model systems. More recently, base editors have been successfully used in cardiac diseases to correct *LMNA* mutations and partially revert pathogenic phenotypes.^22^^,^^23^ For L-CMD, only one pre-clinical study reported the potential of *LMNA*-mRNA repair by spliceosome-mediated RNA *trans*-splicing. However, this gene therapy strategy showed a low-efficiency outcome.^24^ In conclusion, there is still no promising approach for the treatment of L-CMD.

Given the monogenic nature of L-CMD, this study explores the potential of a CRISPR gene editing approach to eliminate the *Lmna* c.745C>T mutation using a mutant allele-specific RNA guide. The goal is to establish a hemizygous state for *Lmna*, aiming to reverse the pathogenic phenotype associated with L-CMD. The study involves testing this approach in different cellular models and the newly generated *Lmna*^*R249W*^ mouse model.

## Results

### Evaluation of CRISPR-Cas9 technology for the elimination of the *Lmna* c.745C>T mutation in mouse embryonic fibroblasts: Molecular characterization and phenotypic outcomes

**Figure 1:**
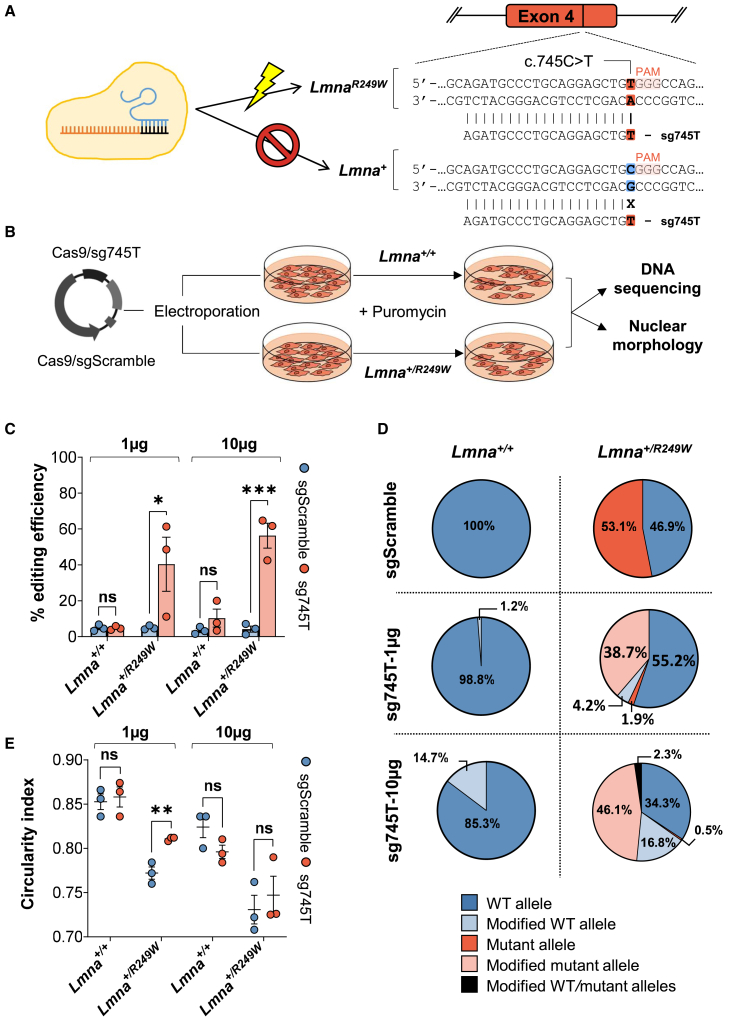
The Cas9/sg745T complex demonstrates allele-specific activity on *Lmna* c.745C>T in MEFs (A) Localization of the sg745T RNA guide in exon 4 of the *Lmna* gene. The nucleotide of the mutation (T = thymine, in red) is at position 745 of the coding sequence, while the nucleotide occupying the same position in the WT allele is highlighted in blue (C = cytosine). The PAM sequence is highlighted. (B) Schematic experimental design for the assessment of activities of the Cas9/sgScramble and Cas9/sg745T complexes in mouse embryonic fibroblasts. Two different genotypes for the *Lmna* gene (*Lmna*^*+/+*^ and *Lmna*^*+/R249W*^) were used for all experiments, and three biological replicates have been performed (*n* = 3 pools). (C) Analysis of CRISPR efficiency at the cellular pool level using TIDE platform after nucleofection of Cas9/sgScramble and Cas9/sg745T complexes. Data are represented as mean ± SD. (D) Percentage of reads for unmodified and modified alleles at the cellular pool level analyzed by CRISPResso2 after nucleofection with Cas9/sgScramble and Cas9/sg745T complexes. (E) Circularity index at the cellular pool level. Data are represented as mean ± SEM. ns, non-significant differences; ∗*p* < 0.05; ∗∗*p* < 0.01; ∗∗∗*p* < 0.001.

Previous studies have demonstrated that CRISPR activity is highly dependent on the sequence complementary to the guide RNA of the CRISPR-Cas complex.^25^ Thus, alteration in just one of the 20 nucleotides of the guide results in a significant reduction in the activity of the Cas9 endonuclease on the target sequence.^26^ This cleavage specificity increases with the proximity of the point mutation to the PAM sequence.^27^ Exploiting this property of the CRISPR system, we aimed to develop a guide RNA that directs Cas9 activity preferentially to the *Lmna* c.745C>T mutant allele with minimal to no effect on the wild-type (WT) allele. Specifically, one guide RNA was designed to contain the cytosine-to-thymidine point mutation found at position 745 in the genomic sequence of the mutant allele (Figure 1A). The activity of the Cas9/sg745T complex was initially evaluated using an *in vitro* endonuclease cleavage assay with exon 4 of the *Lmna* gene, amplified from mouse embryonic fibroblasts (MEFs). In samples derived from MEFs harboring one or two copies of the c.745C>T mutation, two distinct bands were observed, indicating successful cleavage by the Cas9/sg745T complex. In contrast, no cleavage activity was detected in DNA amplified from *Lmna*^*+/+*^ MEFs (Figure S1). Therefore, Cas9/sg745T complex activity is only observed in the presence of the mutant allele, demonstrating the specificity of the complex for the mutant allele containing the cytosine-to-thymine mutation.

To validate our strategy, plasmids expressing Cas9 plus sg745T or a scramble RNA guide were delivered into WT (*Lmna*^*+/+*^) and heterozygous (*Lmna*^*+/R249W*^) MEFs carrying the c.745C>T mutation (Figure 1B). Two DNA amounts (1 and 10 μg) were tested. To determine CRISPR activity, Sanger sequencing data were analyzed using the TIDE platform (Figures 1C and S2). Deletions represented the most frequent indel type in both genotypes (Figure S2). In WT fibroblasts at low DNA concentration (1 μg), both Cas9/sgScramble (4.8% ± 1%) and Cas9/sg745T complexes (4.7 ± 0.6%) induced similar residual CRISPR activity (*p* = 0.46). When the amount of Cas9/sg745T complexes were increased (10 μg) in *Lmna*^*+/+*^ MEFs, this activity increased to 10.3% ± 4.9%, but there were no significant differences compared to its control Cas9/sgScramble (3.4% ± 1.3%, *p* = 0.12). However, significant differences were detected in the heterozygous line for the c.745C>T mutation between Cas9/sg745T and Cas9/sgScramble complexes. In *Lmna*^*+/R249W*^ MEFs, electroporation of 1 μg of Cas9/sg745T plasmid resulted in an editing efficiency significantly higher than that observed for Cas9/sgScramble (40.4% ± 15% vs. 5 ± 0.7%, *p* = 0.04). At higher concentrations (10 μg), the activity of Cas9/sg745T complexes significantly increased to 56.3% ± 6.9%, compared to the activity of 4.2% ± 1.7% detected for Cas9/sgScramble complexes (*p* < 0.001).

These results were further confirmed through amplicon deep sequencing analysis of the target sequence using the CRISPResso2 platform (Figure 1D). In a *Lmna*^*+/+*^ scenario, 100% of reads were obtained for the original WT allele in the control condition (Cas9/sgScramble). When a low concentration (1 μg) of Cas9/sg745T complexes was used, similar results were observed (98.8% ± 1.2% of reads for the original WT allele). When the concentration of Cas9/sg745T complexes was increased to 10 μg, the percentage of unmodified WT allele reads decreased to 85.3% ± 8.5%, while 14.7% ± 6% of the reads corresponded to modified WT sequences. In the case of *Lmna*^*+/R249W*^ cells, the expression of Cas9/sgScramble complexes resulted in similar percentages of reads of WT and mutant sequences. As expected for this control, no reads for either of these two alleles were detected as modified. However, electroporation with 1 μg of Cas9/sg745T complexes led to a high percentage of reads for the modified mutant allele (38.7% ± 7.9% of total sequences). This effect was accompanied by a drastic reduction in reads for the unmodified mutant allele (1.9% ± 1% of the total). It is interesting to note that under these conditions, the percentage of modified WT allele constituted only 4.2% ± 2% of the total reads detected. When high concentrations (10 μg) of Cas9/sg745T complexes were used, the percentage of reads for the modified mutant allele increased even further to 46.1% ± 4.3% of the total. Simultaneously, the percentage of detected reads for the unmodified mutant allele was nearly zero (0.5% ± 0.5% of sequences). Interestingly, a moderate increase in modified WT sequences (16.8% ± 3.5%) was observed under these high conditions. A detailed characterization of Cas9-induced editing revealed multiple distinct indels in both *Lmna*^*+/+*^ and *Lmna*^*+/R249W*^ cells (Figures S3 and S4). As expected, indel diversity was higher in mutant MEFs compared with WT cells and correlated with Cas9/sg745T concentration. The most frequent event was a single-nucleotide deletion (c.743delT). Notably, this alteration, and most of those identified in both WT and mutant MEFs, introduces a premature stop codon (Table S1). All these analyses confirmed the specificity of Cas9/sg745T complexes for the mutant allele and demonstrated that their activity is concentration dependent.

It has been reported that nuclei of cells carrying the c.745C>T mutation exhibited compromised nuclear membrane integrity.^28^ MEFs harboring this variant show significantly lower circularity indices compared to WT cells, indicating altered nuclear morphology (Figure S5). The elimination of the c.745C>T mutation is presumed to restore nuclear morphology. To test this hypothesis, the circularity index of nuclei from WT and *Lmna*^*+/R249W*^ MEFs previously transfected with Cas9/sgScramble and Cas9/sg745T complexes was quantified (Figure 1E). In the case of WT fibroblasts, no significant differences in circularity index were found when using Cas9/sg745T or Cas9/sgScramble complexes at any of the two concentrations used. In contrast, in a heterozygous background, an increase in the circularity of nuclei from cells nucleofected with Cas9/sg745T complexes was observed compared to those nucleofected with Cas9/sgScramble complexes. This phenotype rescue was statistically significant only in the low concentration condition (*p* = 0.003), which, according to the deep sequencing studies, was associated with the highest percentage of unmodified WT allele (53.1% ± 3.3%) and lowest residual unmodified mutant allele (1.9% ± 1% of total reads). These results demonstrated that the specific removal of the *Lmna* c.745C>T mutation improves the nuclear morphology of heterozygous MEFs.

### Study of specificity of CRISPR-Cas9 technology in embryos of the *Lmna*^*R249W*^ mouse model

**Figure 2:**
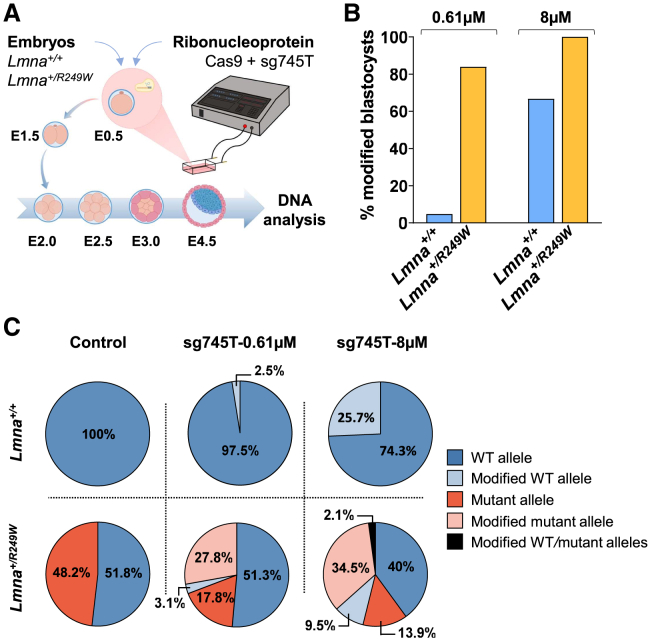
The Cas9/sg745T complex manifests high specificity for the *Lmna* c.745C>T allele in mouse embryos (A) Schematic experimental design for studying the activity of Cas9/sg745T complex in mouse embryos. For all experiments, two different genotypes for the *Lmna* gene (*Lmna*^*+/+*^ and *Lmna*^*+/R249W*^) were used. (B) Percentage of modified blastocysts after the introduction of Cas9/sg745T complexes. (C) Percentage of reads for unmodified and modified alleles analyzed by CRISPResso2 in modified blastocysts after treatment with Cas9/sg745T complexes. In the control condition, 13 *Lmna*^*+/+*^ and 17 *Lmna*^*+/R249W*^ blastocysts were used. In the 0.61 μM condition, 21 *Lmna*^*+/+*^ and 31 *Lmna*^*+/R249W*^ blastocysts were employed. In the 8 μM condition, 27 *Lmna*^*+/+*^ and 21 *Lmna*^*+/R249W*^ blastocysts were used.

One-cell-stage embryos were obtained from matings between *Lmna*^*+/+*^ and *Lmna*^*+/R249W*^ animals and nucleofected with the Cas9 and sg745T as a ribonucleoprotein complex (Figure 2A). Like in previous experiments, two concentrations of the Cas9:sgRNA complex were tested: a low concentration (0.61 μM) and a high concentration (8 μM). Nucleofected embryos were cultured to the blastocyst stage (embryonic day [E] 4.5), at which time genomic DNA was extracted, the target sequence was amplified by PCR, and editing frequency analyzed using Tracking of Indels by Descomposition (TIDE) after Sanger sequencing. A total of 130 blastocysts were analyzed. As shown in Figure 2B, at the low concentration (0.61 μM), only 4.5% of *Lmna*^*+/+*^ blastocysts exhibited modifications (1 of 21), while 83.9% of *Lmna*^*+/R249W*^ blastocysts (26 of 31) contained indels, primarily nucleotide insertions. At the high concentration (8 μM), the modification rate for *Lmna*^*+/R249W*^ blastocysts rose to 100%, with nucleotide insertions remaining the most frequent indel type. Modifications in *Lmna*^*+/+*^ blastocysts also increased significantly, from 4.5% to 66.7%.

To further assess the specificity of the Cas9/sg745T complex for the c.745C>T mutant allele, blastocyst DNA was analyzed by amplicon deep sequencing (Figure 2C). In *Lmna*^*+/+*^ control blastocysts (no CRISPR introduced), 100% of reads corresponded to the WT allele. At 0.61 μM, 97.5% of reads were unmodified WT, with only 2.5% showing indels. At 8 μM, unmodified WT allele reads decreased to 74.3% ± 3.9%, with modified WT reads increasing to 25.7% ± 3.8%. On the other hand, in *Lmna*^*+/R249W*^ control blastocysts, WT and mutant alleles were equally represented (51.8% ± 2.1% vs. 48.2% ± 2.1%). At 0.61 μM, mutant allele reads decreased to 17.8% ± 1.6%, with 27.8% ± 2.5% containing Cas9-induced modifications, while WT allele modifications remained minimal (3.1% ± 1%). At 8 μM, mutant allele modifications increased to 34.5% ± 4.7% and unmodified mutant allele reads further decreased (13.9% ± 1.9%). WT allele modifications also rose to 9.5% ± 5.6%. A detailed analysis of the indels generated upon Cas9 activity in mouse embryos revealed a moderate diversity of variants that correlated with Cas9/sg745T concentration and was consistently higher in mutant alleles than in WT ones (Figure S6). In WT blastocysts, the most frequent indels were missense mutations (c.740A>G, p.E247G and, to a lesser extent, c.746G>A p.R249Q). In contrast, the predominant indel in mutant blastocysts was the c.742_743insT, which generates a premature stop (Table S2). These results confirmed the high specificity of Cas9/sg745T complexes for the c.745C>T allele and revealed a proportional reduction in CRISPR activity targeting the mutant allele as the concentration of CRISPR complexes increased.

### Study of the potential of Cas9/sg745T mediated by AAV9 in an *in vivo*, metabolic context

After establishing the *in vitro* specificity of Cas9/sg745T complex for the mutant *Lmna* c.745C>T allele, we proceeded to validate the CRISPR-mediated strategy to eliminate the mutant allele in the *Lmna*^*R249W*^ murine model. Our initial focus was on *Lmna*^*R249W/R249W*^ mice, which exhibit a severe metabolic phenotype characterized by considerable growth delay, complete absence of hepatic glycogen deposits, reduced adipose tissue, and lowered body temperature. These defects culminate in premature death, with a median survival of 50 days. On the other hand, lamin A/C knockout mice are known to survive no longer than 56 days.^29^ Applying Cas9/sg745T complex therapy in the *Lmna*^*R249W/R249W*^ model is expected to yield the elimination of the mutant allele, which will generate a lamin A/C knockout mouse, which also succumbs prematurely. However, even a modest extension of survival—by up to 6 days, representing a 12% increase, would provide valuable insight into the potential therapeutic effects of this approach and allow for an initial evaluation of its impact.

**Figure 3:**
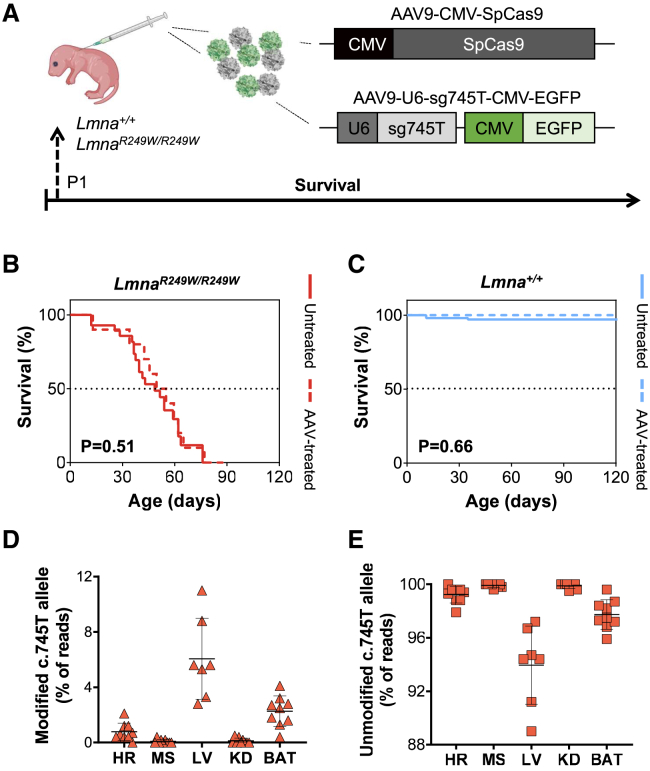
Administration of AAV9-Cas9/sg745T gene therapy does not improve survival in a homozygous background for the R249W mutation (A) Schematic experimental design to evaluate the survival and CRISPR activity after intradermal administration of AAV9-Cas9/sg745T treatment. AAV9-Cas/sg745T was injected into 1-day-old *Lmna*^*R249W/R249W*^ and *Lmna*^*+/+*^ mice. Control mice received no treatment. (B) Kaplan-Meier survival curve of untreated (*n* = 28) and AAV-treated (*n* = 11) *Lmna*^*R249W/R249W*^ mice. (C) Kaplan-Meier survival curve of untreated (*n* = 100) and AAV-treated (*n* = 26) *Lmna*^*+/+*^ mice. (D) Percentage of modified c.745T allele reads in *Lmna*^*R249W/R249W*^ AAV-treated mice (n = 7–9). (E) Percentage of unmodified c.745T allele reads in *Lmna*^*R249W/R249W*^ AAV-treated mice (n = 7–9). The activity of Cas9/sg745T complex was analyzed in different tissues: heart (HR), muscle (MS), liver (LV), kidney (KD), and brown adipose tissue (BAT). Data are represented as mean ± SD.

To investigate the *in vivo* potential of Cas9/sg745T gene editing in this model, infections were performed in 1-day-old neonates of both *Lmna*^*R249W/R249W*^ and *Lmna*^*+/+*^ mice using adeno-associated virus (AAV) serotype 9 (AAV9) vectors (1 × 10^11^ viral genomes of each vector) administered via intradermal injection in the interscapular region (Figure 3A). Two different viral vectors were used: one carrying the Cas9 endonuclease under the CMV promoter (AAV9-CMV-SpCas9) and the other containing the sg745T RNA guide sequence driven by the U6 promoter and the EGFP expression gene under the CMV promoter (AAV9-U6-sg745T-CMV-eGFP). A pilot experiment using the AAV9-U6-sg745T-CMV-eGFP vector confirmed the high infectivity of this AAV in the expected target tissues (skeletal and cardiac muscle, brown adipose tissue, and liver; Figure S7). The impact of this AAV-CRISPR-mediated therapy, referred to as AAV9-Cas9/sg745T or AAV-treated hereafter, on survival and growth was evaluated compared to untreated control groups (untreated *Lmna*^*+/+*^ and *Lmna*^*R249W/R249W*^ mice), referred to as untreated hereafter.

The survival of AAV-treated *Lmna*^*R249W/R249W*^ mice showed a modest, although not statistically significant, increase of 10% compared to untreated, control mice (55 days vs. 50 days, respectively, *p* = 0.51) (Figure 3B). This trend was consistent in both sexes, with AAV-treated males averaging 51 days compared with 42 days in untreated, control (*p* = 0.29) and AAV-treated females averaging 59 days vs. 55 days in untreated, control mice (*p* = 0.89) (Figure S8A). In a WT background (Figure 3C), there was no significant survival difference between AAV-treated and untreated mice (*p* = 0.66).

Interestingly at 21 days of age AAV-treated, *Lmna*^*R249W/R249W*^ mice weighed significantly less than untreated controls (*p* = 0.002) (Figure S8B). However, this difference was no longer apparent by 35 days of age, with AAV-treated *Lmna*^*R249W/R249W*^ mice showing a slight, albeit non-significant, increase in average body weight compared to untreated controls (*p* = 0.20) (Figure S8B). When stratified by sex, the difference in body weight at 21 days of age between AAV-treated *Lmna*^*R249W/R249W*^ and untreated control mice was only significant in males (*p* < 0.01) (Figure S8B). By 35 days post-treatment, there were no significant body weight differences between treated and control mice for either sex (Figure S8B). Overall, these findings indicate that AAV9-Cas9/sg745T therapy has limited impact on the survival or growth of *Lmna* ^*R249W/R249W*^ mice.

The observed 10% survival increase in *Lmna*^*R249W/R249W*^ mice (approaching the 56 days survival in lamin A/C knockout animals) could potentially be attributed to the editing of the two R249W alleles, leading to truncated alleles. To explore this hypothesis, CRISPR activity was analyzed in various tissues (heart, muscle, liver, kidney, and brown adipose tissue) of *Lmna*^*+/+*^ and *Lmna*^*R249W/R249W*^ mice aged 42–90 days post-treatment. Genomic DNA was extracted, and exon 4 of the *Lmna* gene was amplified for NGS analysis. No CRISPR activity was detected in *Lmna*^*+/+*^ mice, while modified sequences were observed in the R249W alleles of homozygous mutant mice (Figure 3D). Thus, a reduction in unmodified mutant alleles was observed in liver and brown adipose tissue of infected *Lmna*^*R249W/R249W*^ mice (Figure 3E). Despite known tropism of AAV9 tropism for cardiac and skeletal muscle, the heart exhibited only a slight reduction in the R249W allele frequency (99.2% ± 0.6%), while muscle was minimally affected (99.9% ± 0.1% R249W allele readings). The highest editing activities occurred in the liver and brown fat, where unmodified mutant allele readings decreased to 93.9% ± 2.9% and 97.7% ± 1.1%, respectively. In kidney, unmodified allele frequencies were 99.9% ± 0.2% consistent with the serotype’s distribution. These data suggest the sg745T guide retains its specificity for the mutated R249W allele *in vivo*, although with greatly reduced activity.

Histopathological analyses of these tissues in treated *Lmna*^*R249W/R249W*^ mice (aged 49–63 days) were compared to 35-day-old untreated controls. No significant differences or abnormalities were observed in the heart, muscle, or kidney between the groups. Although the liver exhibited the highest CRISPR activity, glycogen deposits were absent in both treated and untreated homozygous animals. In brown fat, treated mice displayed a noticeable reduction or absence of multilocular fat vacuoles, unlike the normal tissue morphology seen in untreated animals. Examination of white fat, pancreas, and spleen revealed no abnormalities in white adipose tissue or pancreas. However, lymphoid hypoplasia was detected in 50% of spleens from treated *Lmna*^*R249W/R249W*^ mice, a feature absent in untreated controls.

In summary, AAV9-Cas9/sg745T therapy resulted in a modest, non-significant 10% increase in survival of *Lmna*^*R249W/R249W*^ mice and showed limited CRISPR activity across tissues.

### Effect of AAV9 Cas9/sg745T gene therapy on cardiac function

*Lmna*^*+/R249W*^ mice develop dilated cardiomyopathy, making them an ideal model to assess the impact of AAV-Cas9/sg745T gene therapy on this key pathological feature of L-CMD. Notably, the AAV vectors employed in this study sustained transgene expression in heart for up to 50 weeks post-infection (Figure S9), confirming their suitability as long-term delivery vehicles for this Cas9-mediated therapy. *Lmna* editing efficiency was assessed in various tissues (heart, muscle, liver, kidney, and brown adipose tissue) of AAV-treated *Lmna*^*+/R249W*^ and *Lmna*^*+/+*^ mice. Samples were collected at 5 and 50 weeks of age to evaluate temporal changes. There was no evidence of editing in WT tissues at either time point (Figure S10A). Similarly, *Lmna*^*+/R249W*^ tissues exhibited no indels in the WT allele (Figure S10B). However, indels were detected in the mutant allele of treated *Lmna*^*+/R249W*^ mice (Figure S10B). At 50 weeks of age, the percentage of modified R249W allele reads increased significantly in the heart (from 0.4% ± 0.1% to 1.3% ± 0.3%, *p* = 0.02) and brown adipose tissue (from 0.3% ± 0.1% to 2.0% ± 0.7%, *p* = 0.004) from 5 to 50 weeks of age. In contrast, there was no significant difference in muscle at any time (0.07% ± 0.04% vs. 0.2% ± 0.1%, *p* = 0.23). The liver exhibited the highest allele editing activity but no significant temporal variation (3.7% ± 1.2% at 5 weeks vs. 2.5% ± 1.1% at 50 weeks, *p* = 0.30). Finally, there was minimal editing in kidney with some residual indels at 5 weeks (0.04% ± 0.04%) but not at 50 weeks of age (*p* = 0.27). As observed in MEFs and blastocysts, detailed analysis of Cas9/sg745T-induced indels in target tissues revealed greater indel diversity in mutant alleles and mice compared with WT counterparts (Figures S11 and S12). A positive correlation was also detected between indel diversity and time in mutant alleles and mice, with higher variability observed at 50 weeks than at 5 weeks, confirming the long-term activity of AAV-delivered CRISPR complexes. The most frequent variant in WT alleles was c.740A>G, whereas c.742_743insT predominated in mutant alleles. As predicted (Table S3), the c.740A>G missense mutation results in a p.E247G substitution, while c.742_743insT introduces a premature stop codon at position 253.

To explore the effects of CRISPR activity, lamin A/C protein levels were evaluated in the heart, muscle, liver, and brown adipose tissue of treated and untreated *Lmna*^*+/+*^ and *Lmna*^*+/R249W*^ mice at 50 weeks of age (Figure S13). Untreated *Lmna*^*+/R249W*^ mice showed slightly reduced lamin A/C levels compared to *Lmna*^*+/+*^ controls, with a significant reduction only in the liver (*p* = 0.04). Treated *Lmna*^*+/R249W*^ mice showed no significant changes in lamin A/C expression compared to untreated counterparts, consistent with the modest gene editing levels detected in these tissues.

**Figure 4:**
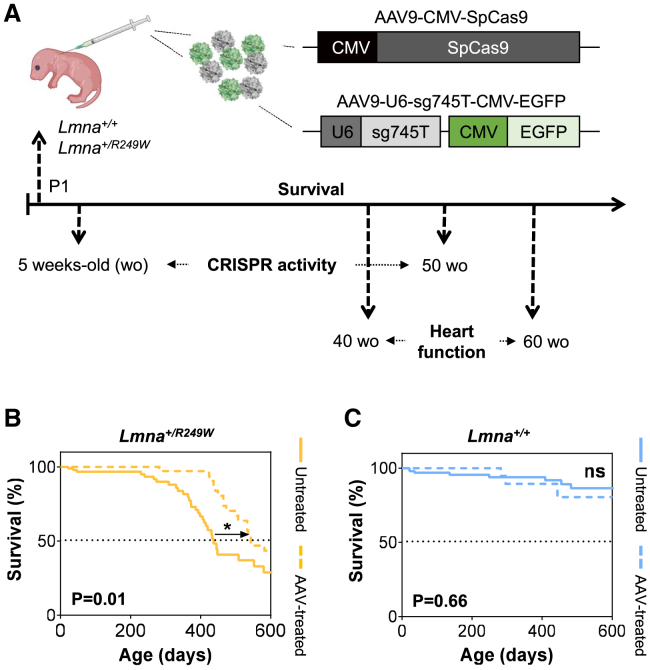
AAV9-Cas9/sg745T treatment increases survival in *Lmna*^*+/R249W*^ mice (A) Schematic experimental design to evaluate the survival and cardiac function after intradermal administration of AAV9-Cas9/sg745T treatment. AAV9-Cas/sg745T was injected into 1-day-old *Lmna*^*+/R249W*^ and *Lmna*^*+/+*^ mice. Control mice received no treatment. (B) Kaplan-Meier survival curve of untreated (*n* = 100) and AAV-treated (*n* = 36) *Lmna*^*+/R249W*^ mice. (C) Kaplan-Meier survival curve of untreated (*n* = 100) and AAV-treated (*n* = 26) *Lmna*^*+/+*^ mice. ns, non-significant differences; ∗*p* < 0.05.

Despite limited editing, AAV9-Cas9/sg745T therapy significantly improved the median survival of *Lmna*^*+/R249W*^ mice, from 437 (untreated) to 543 days (*p* = 0.01, Figure 4). This 24.3% increase in lifespan was observed in males (543 vs. 415 days, *p* = 0.01), but not in females (*p* = 0.71) (Figure S14). AAV-Cas9/sg745T treatment had no impact on survival of *Lmna*^*+/+*^ mice (Figure 4C; Figure S14).

**Figure 5:**
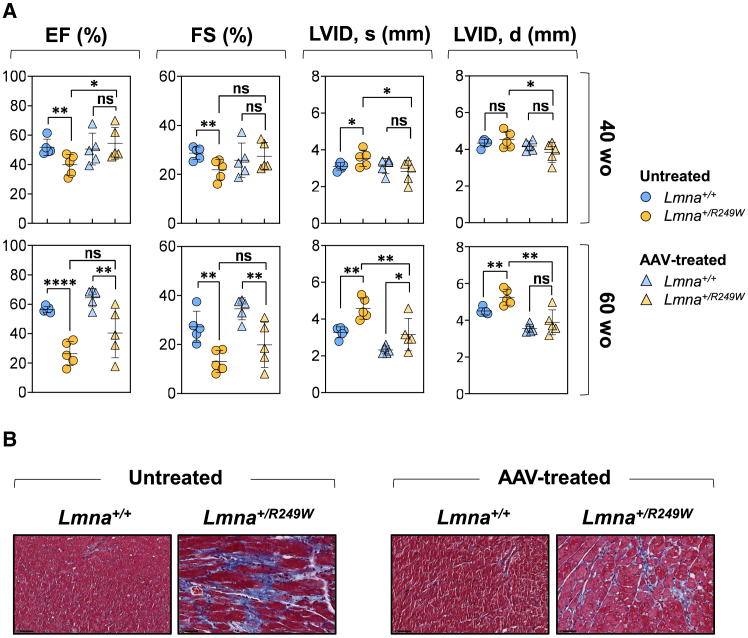
Single dose of AAV9-Cas9/sg745T treatment improves cardiac function in *Lmna*^*+/R249W*^ mice (A) Echocardiographic measurements of LVID, s; LVID, d; EF; and FS at 40 and 60 weeks of age. The echocardiographic study was conducted in untreated (*n* = 5 of each genotype) and AAV-treated (*n* = 5 of each genotype) males. Data are represented as mean ± SD. ns, non-significant differences; ∗*p* < 0.05; ∗∗*p* < 0.01; ∗∗∗∗*p* < 0.0001. (B) Representative images with Masson’s trichrome staining of heart sections from untreated (*n* = 3 of each genotype) and AAV-treated (*n* = 3 of each genotype) males at 50 weeks of age. Scale bars, 50 μm.

Given that *Lmna*^*+/R249W*^ mice develop dilated cardiomyopathy, the impact of AAV9-Cas9/sg745T treatment on cardiac function was assessed at 40 and 60 weeks of age using echocardiography. At 40 weeks of age, treated *Lmna*^*+/R249W*^ mice showed significant improvement in left ventricular end-systolic (LVID, s) and end-diastolic (LVID, d) diameters (*p* = 0.03 and *p* = 0.03, respectively) and ejection fraction (EF, *p* = 0.02), but not for fractional shortening (FS, *p* = 0.06), compared to untreated *Lmna*^*+/R249W*^ mice (Figure 5A). These values were comparable to those of WT treated mice. At 60 weeks of age, treated *Lmna*^*+/R249W*^ mice showed significant smaller ventricular diameters than untreated mice (LVID, s: *p* = 0.01 and LVID, d: *p* = 0.003, Figure 5A) and no significant improvement in EF and FS (*p* = 0.07 and *p* = 0.09, respectively, Figure 5A). This is consistent with progression of dilated cardiomyopathy in treated animals (LVID, s: 3.15 ± 0.88 mm, *p* = 0.04; EF: 40.42% ± 16.81%, *p* = 0.01).

In addition to the echocardiographic studies, a histopathological evaluation of the hearts from AAV9-Cas9/sg745T-treated and untreated animals was conducted. This analysis revealed interstitial fibrosis in the hearts of treated *Lmna*^*+/R249W*^ mice at 50 weeks of age, ranging from mild to severe. Severe fibrosis was observed in 33.3% of treated mice vs. 100% of untreated animals (Figure 5B). Treated WT mice displayed no pathological changes. We also evaluated potential adverse effects associated with AAV-mediated treatment by performing a detailed histopathological analysis of *Lmna*^*+/R249W*^ mice, both AAV-treated and untreated. As summarized in Table S4, no abnormalities were observed in the gastrocnemius muscle, pancreas, or spleen of either group. Age-related changes were noted in kidneys and lungs across both groups. Notably, differences were confined to the liver and adipose tissue: while AAV-treated mice showed normal liver histology, two of three untreated animals displayed anisocytosis and reduced glycogen content. In contrast, both brown and white adipose tissues were normal in untreated mice but exhibited a lipodystrophic phenotype in all AAV-treated animals.

In summary, a single dose of AAV9-Cas9/sg745T therapy extends the lifespan of *Lmna*^*+/R249W*^ mice by 24.3% and partially mitigates cardiac dysfunction, as evidenced by reduced interstitial fibrosis and delayed cardiomyopathy progression. These findings highlight the potential of this gene editing strategy for addressing *LMNA*-related cardiac diseases.

## Discussion

### Efficiency and specificity of Cas9/sg745T in eliminating of the *Lmna* c.745C>T mutation

This study demonstrates the efficiency and specificity of the Cas9/sg745T complex in selectively deleting the pathogenic *Lmna* c.745C>T mutation in various cell types and a mouse model. In *Lmna*^*+/R249W*^ fibroblasts, the Cas9/sg745T achieved editing efficiencies of 40.4% and 56.3% at plasmid concentrations of 1 and 10 μg, respectively (Figure 1C). In *Lmna*^*+/R249W*^ mouse blastocysts, efficiency was even higher, reaching 83.9% and 100% at CRISPR concentrations of 0.61 and 8 μM, respectively (Figure 2B). Although CRISPR efficiency depends on several factors, the RNA guide sequence plays a critical role in the specificity of Cas9 activity.^30^^,^^31^ It has been reported that Cas9 endonuclease may tolerate mismatches at different positions between the guide RNA and the target DNA.^32^^,^^33^ However, the seed region, consisting of the first five to eight nucleotides proximal to the PAM, is essential for initial target recognition and binding.^34^^,^^35^^,^^36^ Accordingly, while single-nucleotide mismatches near the PAM disrupt Cas9 activity, those located in distal positions may still allow cleavage.^32^^,^^33^^,^^35^ The fact that the c.745C>T mutation resides within the seed region adjacent to the PAM may explain the high specificity of Cas9/sg745T complexes for the mutant target.

Another key aspect that impacts both the efficiency and specificity of the CRISPR complex is the dosage of Cas9 and guide RNA components. In *Lmna*^*+/R249W*^ MEFs, low concentrations of Cas9/sg745T complexes produced a high percentage of indels in the mutant allele, with a substantial reduction in unmodified mutant alleles, leaving only 1.9% of reads unmodified (Figure 1D). At higher concentrations, the unmodified mutant allele was nearly eliminated, with only 0.5% of the reads remaining. A similar trend was observed in mouse embryos, where increasing concentrations of CRISPR components led to more embryos showing modifications (Figure 2B). In *Lmna*^*+/R249W*^ blastocysts, the reduction of the unmodified mutant allele was less pronounced (17.8% and 13.9% of reads at low and high concentrations, respectively; Figure 2C). Importantly, at higher concentrations, activity was also detected in the WT allele of murine fibroblasts, with similar effects observed in embryos. This indicates that while Cas9/sg745T complexes demonstrate high specificity at lower doses, specificity decreases as concentrations increase. Previous studies suggest that high concentrations can reduce mutagenesis efficiency,^37^^,^^38^ emphasizing the importance of optimizing CRISPR component levels to balance efficiency and specificity. In our experiments, the dose-dependent nature of CRISPR activity is also evident in its impact on reducing aberrant nuclear morphology, a common cellular defect in L-CMD and other laminopathies. *Lmna*^*+/R249W*^ fibroblasts display irregular nuclear morphology, consistent with prior reports in human and animal models.^24^^,^^39^^,^^40^^,^^41^ Reduction of nuclear defects has been previously demonstrated in other laminopathy models, such as *LMNA* c.1824C>T (p.G608G) in HGPS patients, where editing improved nuclear morphology.^17^ Similarly, Cas9-mediated indels targeting exon 11 of *LMNA* reduced nuclear alterations in *Lmna*^*G609G/G609G*^ mouse fibroblasts and *Lmna*^*+/G608G*^ patient cells.^16^ In this study, deletion of the *Lmna* c.745C>T allele improved nuclear circularity in *Lmna*^*+/R249W*^ fibroblasts (Figure 1E). However, higher CRISPR doses did not enhance phenotypic rescue, likely due to off-target activity in the WT allele. These findings highlight the importance of optimizing CRISPR dosage to balance efficiency and specificity.

An important finding of our study is the characterization of the indel patterns induced by Cas9/sg745T complexes. Comparison across the three experimental models (Table S5) revealed that Cas9-induced indels were largely consistent between blastocysts and tissues—c.742_743insT, p.(253∗) in mutant alleles and c.740A>G, p.E247G in WT alleles—yet differed from those observed in MEFs where the predominant event was c.743delT, p.(263∗). Notably, most indels detected in mutant alleles consisted of single-nucleotide insertions or deletions that introduced premature stop codons, while in-frame mutations resulting from 3-nucleotide-multiple indels were extremely rare. In summary, Cas9/sg745T activity preferentially generates disruptive mutations that specifically abolish expression of the *LMNA* c.745C>T p.R249W variant.

### Differential effects of AAV9-Cas9/sg745T gene therapy in metabolic and cardiac contexts

This study evaluated AAV9-Cas9/sg745T therapy in metabolic and cardiac settings, revealing distinct therapeutic outcomes. In the metabolic context, treatment only provided minimal benefits in *Lmna*^*R249W/R249W*^ animals, extending median survival by just 10% (Figure 3B). Editing efficiency was low across tissues, with indels detected mainly in the liver (6.1%), followed by brown adipose tissue (2.3%), heart (0.8%), and muscle (0.1%) (Figure 3D). These findings align with prior studies in *Lmna*^*G609G/G609G*^ mice treated with AAV9-SaCas9, where editing was highest in the liver (13.6%) and lower in heart (5.3%) and muscle (4.1%), but resulted in a 26.4% survival increase and improved weight gain.^16^ In contrast, *Lmna*^*R249W/R249W*^ mice showed only a slight, non-significant weight increase (Figure S8B) with no restoration of hepatic glycogen stores or white fat deposits. This limited therapeutic effect could result from the low editing efficiency or the intrinsic challenge of editing both mutant alleles. Even with complete editing, elimination of both R249W alleles would result in a *Lmna* knockout, a condition associated with mortality by 56 days of age.^29^ It is important to note that the *Lmna*^*R249W/R249W*^ mouse model does not reproduce the genetic background found in human L-CMD patients, who are heterozygous for the mutation. Nevertheless, it recapitulates several key features of the disease, such as early onset and the fat tissue abnormalities associated with a metabolic phenotype. Our findings using this model highlight the narrow therapeutic window available in this metabolic context and provide valuable insight into the potential challenges that may arise when applying gene therapy to L-CMD patients with advanced disease.

In contrast, AAV9-Cas9/sg745T therapy had a more pronounced impact in the cardiac context, improving survival and mitigating the cardiac phenotype in *Lmna*^*+/R249W*^ mice (Figures 4 and 5). Similar to the homozygous setting, the highest indel levels were detected in the liver. Interestingly, indel frequencies in cardiac and brown adipose tissues increased significantly over time, from 0.4% and 0.3% at 5 weeks post-treatment to 1.3% and 2.0% at 50 weeks, respectively (Figure S10B). These dynamics align with previous studies targeting the *Myh6* R403Q mutation in hypertrophic cardiomyopathy models^42^ where AAV9-SaCas9/sgRNA treatment at comparable doses resulted in low initial indel rates (0.04% and 1.19% at 5 and 30 weeks) in the R403Q allele that increased over time in heart. Notably, higher doses resulted in greater editing efficiencies (3%–5.9%), highlighting a dose-dependent therapeutic effect. Importantly, our cardiac analysis likely underestimated editing efficiency because it included multiple cell populations. Given that cardiomyocytes—the primary targets of AAV9—constitute only 25%–35% of the total cardiac cells,^43^^,^^44^ single-cell sequencing could have yielded more precise measurements. Further analysis of *Lmna* expression at the mRNA level could also clarify reductions in mutant allele expression due to large deletions.

Importantly, no off-target editing was detected in the WT allele of *Lmna*^*+/R249W*^ mice at either 5 or 50 weeks post-treatment (Figure S10B). This aligns with findings in *Myh6*^*+/R403Q*^ animals, where no WT allele activity was observed at 5 weeks, although higher doses resulted in 4% and 9% loss of the WT allele by 30 weeks.^42^ In contrast, our study showed no modifications in the WT allele of treated WT mice at either 5 or 50 weeks of age (Figure S10A), consistent with the absence of off-target effects and normal cardiac function in these animals.

While CRISPR activity did not alter LMNA protein expression levels (Figure S13), AAV9-Cas9/sg745T therapy significantly improved survival in *Lmna*^*+/R249W*^ mice, extending median survival by 24.3% (Figure 4B). By 40 weeks post-treatment, cardiac function improved, including reduced left ventricular telesystolic and telediastolic diameters and rescued EF and FS (Figure 5A). However, by 60 weeks, signs of dilated cardiomyopathy emerged, although they remained less severe than in untreated heterozygous animals. Histopathological analysis confirmed these findings revealing interstitial fibrosis in treated animals, albeit to a lesser extent than in untreated mice (Figure 5B). These results suggest that even low levels of edited cardiac cells provide therapeutic benefits, likely by reducing the mutation burden and enhancing cellular functions. However, the lack of sustained benefits over time may reflect insufficient infection of target cells or suboptimal editing efficiency in infected cells. The absence of a sustained curative effect, even with preventive approaches, mirrors outcomes reported in other studies targeting LMNA-related diseases, regardless of whether CRISPR1.0^15^^,^^16^ or more advanced gene editing strategies were employed.^17^^,^^19^^,^^21^ Optimization of several parameters could enhance long-term efficacy. Dosage is a key factor since Myh6^+/R403Q^ mice treated with AAV9-SaCas9/sgRNA intermediate doses effectively corrected cardiac hypertrophy without inducing dysfunction,^43^ suggesting that careful dose titration could balance efficacy and safety at pre-clinical stages. Editing efficiency could be improved at multiple levels. For instance, extending or decreasing sgRNA length has been proposed to increase CRISPR activity.^45^^,^^46^ We tested sgRNA variants extended by one or two nucleotides relative to the canonical 20-nucleaotide sequence but observed no differences in editing efficiency (data not shown). Enhancing AAV transduction efficiency represents another opportunity. Although the AAV9 vectors employed here achieved robust cardiomyocyte infection (see Figure S7), newer variants—such as MYO-tropic capsids—may further increase infectivity, particularly in cardiac progenitor cells. Alternative delivery platforms, including nanoparticle-based systems, could also enhance CRISPR efficiency, especially if they exhibit low immunogenicity and enable chronic administration for cumulative correction over time. Finally, Cas9 intracellular dynamics likely play a crucial role. Proper Cas9 expression, stability, and nuclear localization are essential parameters to consider in future design iterations.

In summary, AAV9-Cas9/sg745T therapy shows promise in improving cardiac function and survival in *Lmna*^*+/R249W*^ mice but demonstrates limited efficacy in a metabolic context. Comprehensive optimization of vector delivery, editing precision, and tissue-specific targeting will be critical for realizing its full therapeutic potential and long-term efficacy.

### Gene therapy in muscular dystrophies and *LMNA*-associated diseases

CRISPR-based exon deletion therapies have been investigated in other muscular dystrophies, particularly Duchenne muscular dystrophy (DMD). Using Cas9 and RNA guides to target and delete specific exons, these approaches have demonstrated significant therapeutic potential in pre-clinical studies involving murine and canine DMD models.^47^^,^^48^^,^^49^^,^^50^^,^^51^ Despite these advancements, the application of CRISPR-Cas9 delivered via AAVs in skeletal muscle laminopathies had not been explored prior to this study. However, similar gene editing strategies have shown promise in progeria models. For example, Beyret et al. delivered two RNA guides via AAV9 in transgenic *Lmna*^*G609G*^ mice expressing Cas9, successfully reducing progerin and lamin A levels, leading to phenotypic improvements such as enhanced physical appearance, reduced weight loss, and extended survival.^15^ Similarly, Santiago-Fernández et al. used AAV9-Cas9 with a guide RNA targeting exon 11 of *LMNA,* resulting in reduced progerin and lamin A accumulation, improved pathological features, and increased survival rates.^16^

Another promising therapeutic approach involves antisense oligonucleotides (ASOs), short nucleotide sequences designed to modulate mRNA expression. Given that many *LMNA* mutations exert dominant-negative effects, suppressing the mutant transcript presents a potential therapeutic strategy. Lee et al. demonstrated that ASO-mediated suppression of lamin A and progerin in *Lmna*^*G609G*^ mice increased lamin C production, reduced aortic pathology, and extended lifespan.^52^ Similarly, Osorio et al. reported that ASO treatment reduced progerin accumulation and extended survival in progeria models.^53^ In human cells, ASOs have also been used to induce exon skipping in the *LMNA* gene.^54^ Additionally, one study has explored spliceosome-mediated RNA *trans*-splicing as a gene therapeutic strategy in L-CMD. In the *Lmna*^*ΔK32*^ mouse model, this technique partially corrected nuclear defects and increased *LMNA* WT mRNA expression in various tissues. However, the improvements were insufficient to extend lifespan.^24^

Currently, base editors can be considered one of the most promising approaches for treating human diseases caused by dominant-negative mutations, such as those associated with laminopathies. Indeed, they have been successfully used to correct pathogenic mutations and ameliorate disease phenotypes in several studies focused on HGPS,^17^^,^^18^^,^^19^^,^^21^ L-CMD,^20^ and *LMNA*-related cardiac diseases.^22^^,^^23^ Although these approaches have yielded encouraging results, they still face challenges similar to those observed with first-generation CRISPR (CRISPR1.0) approaches. For instance, none have fully reverted the pathological phenotype, even when applied in preventive protocols. Moreover, bystander editing could significantly limit their use. Unfortunately, for the LMNA c.745C>T mutation analyzed in this work, the most frequent in L-CMD, base editing could introduce a pathogenic bystander mutation (LMNA p.R248P), which has been reported in a patient with Emery-Dreifuss muscular dystrophy.^55^ Therefore, further comparative studies will be required to establish whether base editing or CRISPR1.0 provides the most suitable therapeutic strategy for *LMNA* c.745C>T-driven L-CMD.

We believe that the CRISPR-mediated strategy presented here constitutes a robust and promising therapeutic approach for L-CMD. Overall, this study represents a significant advancement in the field of gene therapy for laminopathies, demonstrating for the first time the successful application of CRISPR-Cas9 to specifically target the *Lmna* c.745C>T mutation underlying L-CMD. Together, our results lay a solid foundation for the further optimization and eventual clinical translation of this technology for the treatment of L-CMD and related laminopathies.

## Materials and methods

The details of the resources used in this research, about antibodies, cell culture media, plasmids, reagents, platforms, and software, are listed in Table S6.

### Cell lines

All cell lines were cultured in an incubator at 37°C under an atmosphere of 5% CO_2_ and 95% humidity.

#### Mouse embryonic fibroblasts

MEFs were isolated and immortalized from a genetically engineered mouse model constitutively expressing the *Lmna* c.745C>T, p.R249W mutation, following the standard protocol.^56^ The study utilized several MEF lines, including WT (*Lmna*^*+/+*^), heterozygous (*Lmna*^*+/R249W*^), and homozygous (*Lmna*^*R249W/R249W*^) genotypes. For genotyping and precise allele identification, the mutant *Lmna* c.745C>T allele was noted to contain an additional silent mutation (c.750T>C) and a *loxP* site in the intron between exons 2 and 3. Cells were cultured in Dulbecco’s Modified Eagle Medium ([DMEM], 4.5 g/L glucose) supplemented with 10% fetal bovine serum and 1% penicillin-streptomycin. For nuclear morphology analysis, MEFs were plated in a 96-well glass-bottom black microplate (5,000 cells/well). After 24 h, cells were fixed with methanol (−20°C, 5 min) and stained with Hoechst 33324 (2 μg/mL in PBS, 37°C, 15 min). Nuclear images were acquired using the Cytell Cell Imaging System, and morphology was assessed via circularity index (0–1, where 1 represents a perfect circle).

#### Mouse embryos

Female *Lmna*^*+/+*^ mice aged 7–8 weeks were hormonally stimulated to enhance follicle production. Superovulation was induced by intraperitoneal administration of equine serum gonadotropin followed 48 h later by an injection of human chorionic gonadotropin. After the completion of the final hormonal treatment, the females were paired with *Lmna*^*+/R249W*^ males aged 9–19 weeks. Successful copulation was confirmed the following morning by the presence of a vaginal plug. Subsequently, the females were euthanized by cervical dislocation, and their oviducts were carefully extracted and washed with M2 culture medium. The oviducts were then transferred to fresh M2 medium containing hyaluronidase (300 μg/mL). To retrieve embryos at E0.5 development stage, the ampullary region of the oviducts was mechanically ruptured. The collected embryos underwent multiple washes in M2 medium and transferred to fresh M2 medium to evaluate their viability and fertilization status. Fertilized embryos were subsequently equilibrated in KSOM medium under a layer of LiteOil Global mineral oil to maintain optimal environmental conditions for further development.

### Mouse model *Lmna*^*R249W*^

Our group previously generated a mouse model of L-CMD by introducing a heterozygous R249W mutation into the *Lmna* gene (unpublished data). The mice were maintained on a predominantly C57BL/6J background. Unless otherwise stated, both male and female mice were included in all experiments in proportional numbers.

Mice were housed in the Animal Facility of the Instituto de Salud Carlos III in ventilated polycarbonate cages, which were equipped with environmental enrichment elements. Animals had *ad libitum* access to food and water. Environmental conditions were carefully controlled, with a temperature maintained between 21°C and 23°C, relative humidity between 55% and 65%, and a 12-h light/dark cycle. For genotyping of *Lmna*^*R249W*^ mice, genomic DNA was extracted from ear tissue by adding 500 μL of 50 mM NaOH, followed by incubation at 99°C–100°C until digestion was complete. Then, 100 μL of 1 M Tris-HCl (pH 7.5) was added, and the mixture was centrifuged at maximum speed for 1 min. A region of the intron between exons 2 and 3 of *Lmna* was PCR-amplified using genotyping-forward (Fw) and genotyping-reverse (Rv) primers (Table S7). The WT allele produced a 217-bp band, while the allele carrying the p.R249W mutation (c.745C>T) produced a 283-bp band. All procedures involving animals were approved by the Research and Animal Welfare Ethics Committee of the Community of Madrid (PROEX164-18) and conducted in compliance with Directive 2010/63/EU on the protection of animals used for experimental and scientific purposes, as implemented in Spanish legislation through Royal Decree 53/2013.

### Endonuclease cleavage assay

Genomic DNA from MEFs was extracted using the E.Z.N.A. Tissue DNA Kit. The *Lmna* exon 4 region was amplified by PCR using 100 ng of DNA and the Lmna-Ex3-Fw and Lmna-Ex5-Rv primers (Table S7). PCR products (682 bp) were confirmed by electrophoresis, excised, and purified with the E.Z.N.A. Gel Extraction Kit. DNA quantification was performed using a NanoDrop spectrophotometer (Thermo Fisher Scientific, Waltham, MA, USA). For Cas9/sg745T ribonucleoprotein (RNP) complex formation, Alt-R CRISPR-Cas9 crRNA and tracrRNA (50 ng each) were incubated at a 1:1 ratio for 5 min at 95°C. The crRNA:tracrRNA complex was then mixed with Cas9 protein and 1× Cas9 buffer (5× stock: 200 mM HEPES, 1 M NaCl, 50 mM MgCl_2_, 1 mM EDTA, pH 6.5) and incubated for 5 min at room temperature.

Each sample underwent two reactions: (1) a digestion reaction containing 100 ng of purified PCR product, 100 ng of Cas9 endonuclease, 100 ng of sg745T RNA guide, 2 μL of Cas9 buffer, and nuclease-free water to a final volume of 10 μL and (2) an undigested control reaction identical to the first but without the Cas9/sg745T complex. Reactions were incubated at 37°C for 6 h and then inactivated at 65°C for 10 min. Digestion products were analyzed by electrophoresis.

### Generation and introduction of CRISPR machinery into mouse cells

The RNA guide was designed to specifically target the c.745C>T mutation in exon 4 of the *Lmna* gene using the Breaking-Cas design tool (https://bioinfogp.cnb.csic.es/tools/breakingcas/).^57^

#### Electroporation of mouse embryonic fibroblasts

The sgRNAs, sg745T and sgScramble (Table S7), were cloned into the pSpCas9(BB)-2A-Puro vector (pX459), which contains the Cas9 endonuclease and a puromycin resistance gene. The pX459 vector was obtained from Feng Zhang’s group.^58^ A total of 1 × 10^6^ MEFs were nucleofected with either pX459-Cas9/sg745T or pX459-Cas9/sgScramble at low (1 μg) or high (10 μg) doses using the NEPA21 electroporator (NepaGene), following the manufacturer’s recommendations and the conditions described in Table S8. After 48 h, puromycin (2 μg/mL) was added for 3 days to enrich successfully transfected cells. The enriched pools were subsequentially expanded for further analysis.

#### Electroporation of mouse embryos

Mouse embryos were divided into two groups: a control group (no electroporation) and an electroporated group (CRISPR machinery introduced). For nucleofection, crRNA and tracrRNA were mixed in a 1:1 ratio to form the sg745T RNA guide and pre-incubated for 5 min at 95°C. The guide was then incubated with the Cas9 protein in Opti-MEM medium for 10 min at 37°C to generate the Cas9/sg745T ribonucleoprotein complex. Embryos were nucleofected with Cas9/sg745T complexes at low (0.61 μM Cas9 endonuclease, 1.83 μM crRNA:tracrRNA) or high (8 μM Cas9 endonuclease, 24 μM crRNA:tracrRNA) doses using the NEPA21 electroporator under the conditions described in Table S9. Immediately after electroporation, zygotes were transferred to KSOM medium and incubated at 37°C with 5% CO_2_ and 95% humidity. After 24 h, embryos reaching the two-cell stage (E1.5) were selected. Both control and electroporated embryos were cultured until the blastocyst stage (E4.5), at which point they were collected for genomic DNA extraction and CRISPR efficiency analysis.

### Genomic DNA sequencing and CRISPR activity analysis

CRISPR-induced modifications in exon 4 of the *Lmna* gene were identified using Illumina NGS and Sanger sequencing at the Genomic Unit of Instituto de Salud Carlos III. DNA was extracted from MEFs and mouse tissues using a commercial kit, while blastocyst DNA was isolated by incubating samples in 17 μL of 50 mM NaOH (95°C, 5 min), followed by neutralization with 1.7 μL of 1 M Tris (pH 8) and overnight incubation at room temperature. Illumina adapters were added to the Cas9-targeted region via PCR using DeepSeq-Fw and DeepSeq-Rv primers (Table S7), followed by a second PCR to incorporate sample-specific indices. FASTQ reads were analyzed using CRISPResso2, applying a 20-bp quantification window (reduced to 10 bp for detailed indel analyses) to identify and quantify small insertions and deletions. For estimating indel frequencies in cells and tissues, only variants with frequencies greater than 1% were considered. No frequency threshold was applied for the detailed characterization of individual indels. Sanger sequencing was performed on all pools and blastocysts using DeepSeq-Fw and DeepSeq-Rv primers. Sequences were analyzed with TIDE to detect genome size modifications post-editing.

### Production and administration of AAV9 viruses

AAV plasmids carrying sg745T RNA guide were constructed by VectorBuilder. Two plasmids were designed: AAV9-U6-sg745T_CMV-EGFP, containing the sg745T guide under the U6 promoter and an EGFP transgene driven by a CMV promoter, and pX551-CMV-SpCas9, expressing *Streptococcus pyogenes* Cas9 under the CMV promoter (obtained from Alex Hewitt’s group via Addgene). AAV9 vectors were produced by triple transient transfection of HEK 293 cells followed by iodixanol gradient purification as previously described.^59^ One-day-old *Lmna*^*+/+*^, *Lmna*^*+/R249W*^, and *Lmna*^*R249W/R249W*^ mice received a single intradermal injection (1 × 10^11^ viral genomes per vector) in the interscapular region using 31G insulin syringes, as previously described.^60^ Control groups remained untreated.

### Phenotypic characterization of mice

#### Body and heart weight

Body weight was recorded weekly from weaning (3 weeks old). After CO_2_ euthanasia, hearts were excised and weighed using a precision balance. Heart weight was normalized to tibia length or body weight.

#### Transthoracic echocardiography

Mice were anesthetized with inhaled isoflurane (2% induction, 1.5% maintenance) while heart rate, respiration, and temperature were monitored. Positioned supine on a heated platform, the parasternal long-axis view was acquired using a Vevo2100 ultrasound system (40-MHz probe, VisualSonics). Two-dimensional and M-mode images were obtained and analyzed blindly using Vevo LAB 5.6.1.

#### Histological analysis

Organs (heart, gastrocnemius muscle, liver, pancreas, spleen, kidney, and brown and white adipose tissues) were fixed in 4% formaldehyde, embedded in paraffin, sectioned (4–6 μm), and stained with hematoxylin and eosin. Longitudinal heart sections were also stained with Masson’s trichrome. Images were acquired using a 3DHistech Mirax scanner and analyzed with NDP.view2.

#### Protein expression analysis

Tissues were frozen (−80°C), mechanically homogenized with a Precellys system (Thermo Fisher) in SDS lysis buffer, and incubated on ice. Lysates were centrifuged (4°C, maximum speed, 30 min), and protein concentration was measured via NanoDrop. Equal protein amounts were resolved on polyacrylamide gels, transferred to nitrocellulose membranes (Trans-Blot Turbo, Bio-Rad), and blocked in 5% milk (PBS-Tween). Membranes were incubated overnight at 4°C with primary antibodies (Table S6), washed, and incubated with secondary antibodies (1 h, room temperature). Protein bands were detected using enhanced chemiluminescence and imaged with Amersham ImageQuant 800. Band intensity was quantified with ImageJ.

#### EGFP immunohistochemistry

One week after infection with AAV9-U6-sg745T-CMV-eGFP, mice were sacrificed, and tissues were fixed in 4% formaldehyde. Samples were subsequently processed by the Histopathology Unit of the National Cancer Research Center. Fixed tissues were embedded in paraffin, sectioned at 4–6 μm using a microtome, and subjected to immunohistochemistry with an anti-EGFP antibody following standard protocols. Stained sections were imaged using the 3DHistech Mirax scanner.

#### ARN isolation and quantitative reverse-transcription PCR

Total RNA was extracted from approximately 30 mg of tissue using 1 mL of TRIzol and mechanical homogenization in a Precellys system (Thermo Fisher Scientific). Following phase separation with chloroform and centrifugation (12,000 × *g*, 30 min, 4°C), RNA was precipitated with isopropanol, washed twice with 75% ethanol, air-dried, and resuspended in Milli-Q water. For cDNA synthesis, 1 μg of total ARN was reverse-transcribed using oligo(dT)20 primers and SuperScript III Reverse Transcriptase (Thermo Fisher Scientific) following the manufacturer’s instructions. Gene expression of Cas9 and EGFP was quantified by qPCR using Fast SYBR Green Master Mix on a QuantStudio 3 (Thermo Fisher Scientific). Reactions were performed in triplicate with gene-specific primers (Table S7). Relative expression was determined using 2ˆ–ΔΔCt method. GAPDH of non-treated mice was used as the reference gene for data normalization.

### Statistical analysis

All analyses and graphs were generated using GraphPad Prism 8.0 software. Data are presented as mean ± SD, with *n* indicating biological replicates. Significance was set as *p* < 0.05. Statistical significance between two experimental groups was determined by one-tailed, unpaired Student’s *t* test. Survival was analyzed using Kaplan-Meier survival curves analyzed via Mantel-Cox log rank test.

## Data and code availability

The data supporting the findings of this study are available within the article and its supplemental information.

## Acknowledgments

We thank Ángel Zaballos, from the Genomics Unit from ISCIII, for carrying out the sequencing and the loan of indices. We want to acknowledge Raquel del Cerro, Javier Esteban, and Elena Velardo, members of the Animal Facility from ISCIII, for all the help and attention dedicated to our *in vivo* experiments. We would also like to thank Patricia González, member of the Histopathology Unit from CNIO, for processing and staining of samples. We acknowledge master thesis student Fernando Gómez García for his support in the performance of MEFs experiments.

This research was supported by grants of Fundación Andrés Marcio, niños contra la laminopatía (TVP 259/19 to I.P.d.C.), Acción Estratégica en Salud Intramural (ISCIII, PI20CIII/00038 and PI23CIII/00041 to I.P.d.C.), Fondo Investigación Sanitaria-FIS-(PI21/00094) co-funded by the 10.13039/501100000780European Union, Fundació Bosch i Aymerichand (to G.S.-B.), and Cure CMD Request for Applications (RFA), International Research Grants in Congenital Muscular Dystrophy (to I.P.d.C.).

## Author contributions

D.G.-D. acquired, analyzed, and interpreted most of the data and drafted/edited the manuscript; C.E. assisted with several *in vivo* experiments and embryo experiments; I.H. and B.V.-M. performed the echocardiography; I.H. assisted in the extraction of genomic DNA; S.C. and G.S.-B. conducted the analysis of echocardiography; A.M. and S.A.-Q. contributed to the experiments aimed at characterizing indels; A.d.M.-I. performed the histopathological study; M.S.-E. was responsible for the production of the adeno-associated virus; I.P.d.C. developed the study concept, obtained funding, coordinated and analyzed the experimental activities, and drafted/edited the manuscript. All authors have read and approved the final manuscript.

## Declaration of interests

The authors declare no conflict of interest. The funders were not involved in the study design, data collection, analysis, interpretation of data, manuscript preparation, or decision to publish the results.

## Declaration of generative AI and AI-assisted technologies in the writing process

During the preparation of this work the authors used AI-based tools for editing purposes, including grammar correction, clarity improvement, and structural refinement. After using this tool, the authors reviewed and edited the content as needed and take full responsibility for the content of the publication.

## Supplemental information

Document S1. Figures S1–S14 and Tables S1–S9

Document S2. Article plus supplemental information

## References

[1] Quijano-Roy S., Mbieleu B., Bönnemann C.G., Jeannet P.-Y., Colomer J., Clarke N.F., Cuisset J.-M., Roper H., De Meirleir L., D’Amico A. (2008). De novo LMNA mutations cause a new form of congenital muscular dystrophy. Ann. Neurol..

[2] Pasqualin L.M.A., Reed U.C., Costa T.V.M.M., Quedas E., Albuquerque M.A.V., Resende M.B.D., Rutkowski A., Chadi G., Zanoteli E. (2014). Congenital muscular dystrophy with dropped head linked to the LMNA gene in a Brazilian cohort. Pediatr. Neurol..

[3] Tan D., Yang H., Yuan Y., Bonnemann C., Chang X., Wang S., Wu Y., Wu X., Xiong H. (2015). Phenotype-Genotype Analysis of Chinese Patients with Early-Onset LMNA-Related Muscular Dystrophy. PLoS One.

[4] Jędrzejowska M., Potulska-Chromik A., Gos M., Gambin T., Dębek E., Rosiak E., Stępień A., Szymańczak R., Wojtaś B., Gielniewski B. (2021). Floppy infant syndrome as a first manifestation of LMNA-related congenital muscular dystrophy. Eur. J. Paediatr. Neurol..

[5] Ben Yaou R., Yun P., Dabaj I., Norato G., Donkervoort S., Xiong H., Nascimento A., Maggi L., Sarkozy A., Monges S. (2021). International retrospective natural history study of LMNA-related congenital muscular dystrophy. Brain Commun..

[6] Shin J.-Y., Worman H.J. (2022). Molecular Pathology of Laminopathies. Annu. Rev. Pathol..

[7] Bertrand A.T., Brull A., Azibani F., Benarroch L., Chikhaoui K., Stewart C.L., Medalia O., Ben Yaou R., Bonne G. (2020). Lamin A/C Assembly Defects in LMNA-Congenital Muscular Dystrophy Is Responsible for the Increased Severity of the Disease Compared with Emery-Dreifuss Muscular Dystrophy. Cells.

[8] Cesar S., Coll M., Fiol V., Fernandez-Falgueras A., Cruzalegui J., Iglesias A., Moll I., Perez-Serra A., Martínez-Barrios E., Ferrer-Costa C. (2023). LMNA-related muscular dystrophy: Identification of variants in alternative genes and personalized clinical translation. Front. Genet..

[9] Fan Y., Tan D., Song D., Zhang X., Chang X., Wang Z., Zhang C., Chan S.H.S., Wu Q., Wu L. (2021). Clinical spectrum and genetic variations of LMNA-related muscular dystrophies in a large cohort of Chinese patients Genotype-phenotype correlations. J. Med. Genet..

[10] Cesar S., Campuzano O., Cruzalegui J., Fiol V., Moll I., Martínez-Barrios E., Zschaeck I., Natera-De Benito D., Ortez C., Carrera L. (2023). Characterization of cardiac involvement in children with LMNA-related muscular dystrophy. Front. Cell Dev. Biol..

[11] Macquart C., Ben Yaou R., Muchir A., Wahbi K., Bonne G. (2016). Clinical features and therapeutic strategies for managing the striated muscle laminopathies. Expert Opin. Orphan Drugs.

[12] Cabral W.A., Tavarez U.L., Beeram I., Yeritsyan D., Boku Y.D., Eckhaus M.A., Nazarian A., Erdos M.R., Collins F.S. (2021). Genetic reduction of mTOR extends lifespan in a mouse model of Hutchinson-Gilford Progeria syndrome. Aging Cell.

[13] Balmus G., Larrieu D., Barros A.C., Collins C., Abrudan M., Demir M., Geisler N.J., Lelliott C.J., White J.K., Karp N.A. (2018). Targeting of NAT10 enhances healthspan in a mouse model of human accelerated aging syndrome. Nat. Commun..

[14] Muchir A., Kim Y.J., Reilly S.A., Wu W., Choi J.C., Worman H.J. (2013). Inhibition of extracellular signal-regulated kinase 1/2 signaling has beneficial effects on skeletal muscle in a mouse model of Emery-Dreifuss muscular dystrophy caused by lamin A/C gene mutation. Skelet. Muscle.

[15] Beyret E., Liao H.-K., Yamamoto M., Hernandez-Benitez R., Fu Y., Erikson G., Reddy P., Izpisua Belmonte J.C. (2019). Single-dose CRISPR–Cas9 therapy extends lifespan of mice with Hutchinson–Gilford progeria syndrome. Nat. Med..

[16] Santiago-Fernández O., Osorio F.G., Quesada V., Rodríguez F., Basso S., Maeso D., Rolas L., Barkaway A., Nourshargh S., Folgueras A.R. (2019). Development of a CRISPR/Cas9-based therapy for Hutchinson–Gilford progeria syndrome. Nat. Med..

[17] Koblan L.W., Erdos M.R., Wilson C., Cabral W.A., Levy J.M., Xiong Z.-M., Tavarez U.L., Davison L.M., Gete Y.G., Mao X. (2021). In vivo base editing rescues Hutchinson-Gilford progeria syndrome in mice. Nature.

[18] Gete Y.G., Koblan L.W., Mao X., Trappio M., Mahadik B., Fisher J.P., Liu D.R., Cao K. (2021). Mechanisms of angiogenic incompetence in Hutchinson-Gilford progeria via downregulation of endothelial NOS. Aging Cell.

[19] Whisenant D., Lim K., Revêchon G., Yao H., Bergo M.O., Machtel P., Kim J.-S., Eriksson M. (2022). Transient expression of an adenine base editor corrects the Hutchinson-Gilford progeria syndrome mutation and improves the skin phenotype in mice. Nat. Commun..

[20] Wang H., Krause A., Escobar H., Müthel S., Metzler E., Spuler S. (2022). LMNA Co-Regulated Gene Expression as a Suitable Readout after Precise Gene Correction. Int. J. Mol. Sci..

[21] Abutaleb N.O., Gao X.D., Bedapudi A., Choi L., Shores K.L., Kennedy C., Duby J.E., Cao K., Liu D.R., Truskey G.A. (2025). Adenine base editing rescues pathogenic phenotypes in tissue engineered vascular model of Hutchinson-Gilford progeria syndrome. APL Bioeng..

[22] Yang L., Liu Z., Sun J., Chen Z., Gao F., Guo Y. (2024). Adenine base editor-based correction of the cardiac pathogenic Lmna c.1621C > T mutation in murine hearts. J. Cell Mol. Med..

[23] Caravia X.M., Hayashi B., Li H., Gan P., Alzhanov D., Tan W., Chen K., McAnally J.R., Xu L., Liu N., Olson E.N. (2025). Precise gene editing of pathogenic Lamin A mutations corrects cardiac disease. Proc. Natl. Acad. Sci. USA.

[24] Azibani F., Brull A., Arandel L., Beuvin M., Nelson I., Jollet A., Ziat E., Prudhon B., Benkhelifa-Ziyyat S., Bitoun M. (2018). Gene Therapy via Trans-Splicing for LMNA-Related Congenital Muscular Dystrophy. Mol. Ther. Nucleic Acids.

[25] Hsu P.D., Scott D.A., Weinstein J.A., Ran F.A., Konermann S., Agarwala V., Li Y., Fine E.J., Wu X., Shalem O. (2013). DNA targeting specificity of RNA-guided Cas9 nucleases. Nat. Biotechnol..

[26] Doench J.G., Fusi N., Sullender M., Hegde M., Vaimberg E.W., Donovan K.F., Smith I., Tothova Z., Wilen C., Orchard R. (2016). Optimized sgRNA design to maximize activity and minimize off-target effects of CRISPR-Cas9. Nat. Biotechnol..

[27] Josephs E.A., Kocak D.D., Fitzgibbon C.J., Mcmenemy J., Gersbach C.A., Marszalek P.E. (2015). Structure and specificity of the RNA-guided endonuclease Cas9 during DNA interrogation, target binding and cleavage. Nucleic Acids Res..

[28] Steele-Stallard H.B., Pinton L., Sarcar S., Ozdemir T., Maffioletti S.M., Zammit P.S., Tedesco F.S. (2018). Modeling Skeletal Muscle Laminopathies Using Human Induced Pluripotent Stem Cells Carrying Pathogenic LMNA Mutations. Front. Physiol..

[29] Sullivan T., Escalante-Alcalde D., Bhatt H., Anver M., Bhat N., Nagashima K., Stewart C.L., Burke B. (1999). Loss of A-type lamin expression compromises nuclear envelope integrity leading to muscular dystrophy. J. Cell Biol..

[30] Kouranova E., Forbes K., Zhao G., Warren J., Bartels A., Wu Y., Cui X. (2016). CRISPRs for optimal targeting: Delivery of CRISPR components as DNA, RNA, and protein into cultured cells and single-cell embryos. Hum. Gene Ther..

[31] O’Geen H., Yu A.S., Segal D.J. (2015). How specific is CRISPR/Cas9 really?. Curr. Opin. Chem. Biol..

[32] Cong L., Ran F.A., Cox D., Lin S., Barretto R., Habib N., Hsu P.D., Wu X., Jiang W., Marraffini L.A., Zhang F. (2013). Multiplex Genome Engineering Using CRISPR/Cas Systems. Science.

[33] Fu Y., Foden J.A., Khayter C., Maeder M.L., Reyon D., Joung J.K., Sander J.D. (2013). High-frequency off-target mutagenesis induced by CRISPR-Cas nucleases in human cells. Nat. Biotechnol..

[34] Semenova E., Jore M.M., Datsenko K.A., Semenova A., Westra E.R., Wanner B., Van Der Oost J., Brouns S.J.J., Severinov K. (2011). Interference by clustered regularly interspaced short palindromic repeat (CRISPR) RNA is governed by a seed sequence. Proc. Natl. Acad. Sci. USA.

[35] Singh D., Sternberg S.H., Fei J., Doudna J.A., Ha T. (2016). Real-time observation of DNA recognition and rejection by the RNA-guided endonuclease Cas9. Nat. Commun..

[36] Wu X., Scott D.A., Kriz A.J., Chiu A.C., Hsu P.D., Dadon D.B., Cheng A.W., Trevino A.E., Konermann S., Chen S. (2014). Genome-wide binding of the CRISPR endonuclease Cas9 in mammalian cells. Nat. Biotechnol..

[37] Ran F.A., Hsu P.D., Lin C.Y., Gootenberg J.S., Konermann S., Trevino A.E., Scott D.A., Inoue A., Matoba S., Zhang Y., Zhang F. (2013). Double nicking by RNA-guided CRISPR Cas9 for enhanced genome editing specificity. Cell.

[38] Ren X., Yang Z., Xu J., Sun J., Mao D., Hu Y., Yang S.J., Qiao H.H., Wang X., Hu Q. (2014). Enhanced specificity and efficiency of the CRISPR/Cas9 system with optimized sgRNA parameters in Drosophila. Cell Rep..

[39] Desgrouas C., Varlet A.-A., Dutour A., Galant D., Merono F., Bonello-Palot N., Bourgeois P., Lasbleiz A., Petitjean C., Ancel P. (2020). Unraveling LMNA Mutations in Metabolic Syndrome: Cellular Phenotype and Clinical Pitfalls. Cells.

[40] Gómez-Domínguez D., Epifano C., de Miguel F., Castaño A.G., Vilaplana-Martí B., Martín A., Amarilla-Quintana S., Bertrand A.T., Bonne G., Ramón-Azcón J. (2020). Consequences of lmna exon 4 mutations in myoblast function. Cells.

[41] Raharjo W.H., Enarson P., Sullivan T., Stewart C.L., Burke B. (2001). Nuclear envelope defects associated with LMNA mutations cause dilated cardiomyopathy and Emery-Dreifuss muscular dystrophy. J. Cell Sci..

[42] Reichart D., Newby G.A., Wakimoto H., Lun M., Gorham J.M., Curran J.J., Raguram A., DeLaughter D.M., Conner D.A., Marsiglia J.D.C. (2023). Efficient in vivo genome editing prevents hypertrophic cardiomyopathy in mice. Nat. Med..

[43] Bergmann O., Zdunek S., Felker A., Salehpour M., Alkass K., Bernard S., Sjostrom S.L., Szewczykowska M., Jackowska T., Dos Remedios C. (2015). Dynamics of Cell Generation and Turnover in the Human Heart. Cell.

[44] Nag A.C. (1980). Study of non-muscle cells of the adult mammalian heart: a fine structural analysis and distribution. Cytobios.

[45] Fu Y., Sander J.D., Reyon D., Cascio V.M., Joung J.K. (2014). Improving CrIsPr-Cas nuclease specificity using truncated guide rNAs. Nat. Biotechnol..

[46] Matson A.W., Hosny N., Swanson Z.A., Hering B.J., Burlak C. (2019). Optimizing sgRNA length to improve target specificity and efficiency for the GGTA1 gene using the CRISPR/Cas9 gene editing system. PLoS One.

[47] Amoasii L., Long C., Li H., Mireault A.A., Shelton J.M., Sanchez-Ortiz E., McAnally J.R., Bhattacharyya S., Schmidt F., Grimm D. (2017). Single-cut genome editing restores dystrophin expression in a new mouse model of muscular dystrophy. Sci. Transl. Med..

[48] Amoasii L., Hildyard J.C.W., Li H., Sanchez-Ortiz E., Mireault A., Caballero D., Harron R., Stathopoulou T.-R., Massey C., Shelton J.M. (2018). Gene editing restores dystrophin expression in a canine model of Duchenne muscular dystrophy. Science.

[49] Karri D.R., Zhang Y., Chemello F., Min Y.L., Huang J., Kim J., Mammen P.P.A., Xu L., Liu N., Bassel-Duby R., Olson E.N. (2022). Long-term maintenance of dystrophin expression and resistance to injury of skeletal muscle in gene edited DMD mice. Mol. Ther. Nucleic Acids.

[50] Min Y.L., Li H., Rodriguez-Caycedo C., Mireault A.A., Huang J., Shelton J.M., McAnally J.R., Amoasii L., Mammen P.P.A., Bassel-Duby R., Olson E.N. (2019). CRISPR-Cas9 corrects Duchenne muscular dystrophy exon 44 deletion mutations in mice and human cells. Sci. Adv..

[51] Zhang Y., Li H., Nishiyama T., McAnally J.R., Sanchez-Ortiz E., Huang J., Mammen P.P.A., Bassel-Duby R., Olson E.N. (2022). A humanized knockin mouse model of Duchenne muscular dystrophy and its correction by CRISPR-Cas9 therapeutic gene editing. Mol. Ther. Nucleic Acids.

[52] Lee J.M., Nobumori C., Tu Y., Choi C., Yang S.H., Jung H.J., Vickers T.A., Rigo F., Bennett C.F., Young S.G., Fong L.G. (2016). Modulation of LMNA splicing as a strategy to treat prelamin A diseases. J. Clin. Investig..

[53] Osorio F.G., Navarro C.L., Cadiñanos J., López-Mejía I.C., Quirós P.M., Bartoli C., Rivera J., Tazi J., Guzmán G., Varela I. (2011). Splicing-directed therapy in a new mouse model of human accelerated aging. Sci. Transl. Med..

[54] Scharner J., Figeac N., Ellis J.A., Zammit P.S. (2015). Ameliorating pathogenesis by removing an exon containing a missense mutation: a potential exon-skipping therapy for laminopathies. Gene Ther..

[55] Vytopil M., Benedetti S., Ricci E., Galluzzi G., Dello Russo A., Merlini L., Boriani G., Gallina M., Morandi L., Politano L. (2003). Mutation analysis of the lamin A/C gene (LMNA) among patients with different cardiomuscular phenotypes. J. Med. Genet..

[56] Tan Y.S., Lei Y.L. (2019). Chapter 7 Generation and Culture of Mouse Embryonic Fibroblasts. Methods Mol. Biol..

[57] Oliveros J.C., Franch M., Tabas-Madrid D., San-León D., Montoliu L., Cubas P., Pazos F. (2016). Breaking-Cas—interactive design of guide RNAs for CRISPR-Cas experiments for ENSEMBL genomes. Nucleic Acids Res..

[58] Ran F.A., Hsu P.D., Wright J., Agarwala V., Scott D.A., Zhang F. (2013). Genome engineering using the CRISPR-Cas9 system. Nat. Protoc..

[59] Lahey H.G., Webber C.J., Golebiowski D., Izzo C.M., Horn E., Taghian T., Rodriguez P., Batista A.R., Ellis L.E., Hwang M. (2020). Pronounced Therapeutic Benefit of a Single Bidirectional AAV Vector Administered Systemically in Sandhoff Mice. Mol. Ther..

[60] Ding J., Lin Z.Q., Jiang J.M., Seidman C.E., Seidman J.G., Pu W.T., Wang D.Z. (2016). Preparation of rAAV9 to Overexpress or Knockdown Genes in Mouse Hearts. J. Vis. Exp..

