# Supplementary material for: CRISPR-mediated targeting of the LMNA c.745C>T mutation enhances survival and cardiac function in congenital muscular dystrophy
Source: Mol Ther Adv. 2025 Dec 26;34(1):201653. doi: 10.1016/j.omta.2025.201653 (PMC13370164; doi:10.1016/j.omta.2025.201653)
Supplement: Document S1. Figures S1–S14 and Tables S1–S9 [file mmc1.pdf]

OMTA, Volume *34*

Supplemental information

CRISPR-mediated targeting of the *LMNA* c.745C>T mutation enhances survival and cardiac function in congenital muscular dystrophy

Déborah Gómez-Domínguez, Carolina Epifano, Iván Hernández, Borja Vilaplana-Martí, Alberto Martín, Sandra Amarilla-Quintana, Sergi Cesar, Antonio de Molina-Iracheta, Miguel Sena-Esteves, Georgia Sarquella-Brugada, and Ignacio Pérez de Castro

FIGURE S1

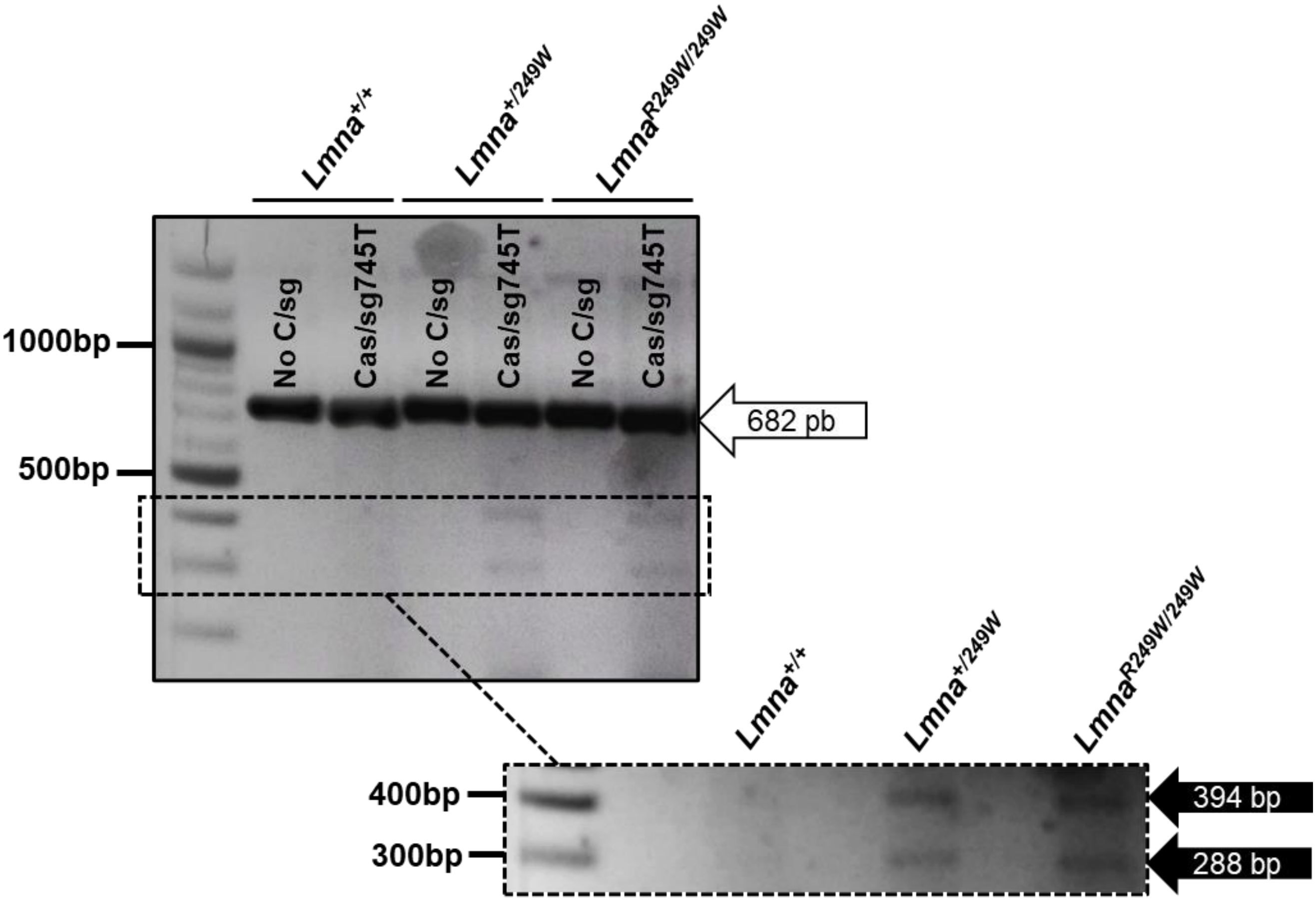

Figure S1. *In vitro* assessment of Cas9/sg745T complex activity in presence of the *Lmna* c.745C>T mutation. Exon 4 of the *Lmna* gene, was amplified by PCR, resulting in a 682 bp fragment. After incubating the PCR product with and without the Cas9/sg745T complex, the reaction outcome was visualized on an agarose gel. Genomic DNA used is derived from embryonic mouse fibroblasts of three different genotype for the *Lmna* gene (WT, heterozygous and homozygous for the c.745C>T mutation). In all cases, two conditions were employed: undigested ("No C/sg": not incubated with Cas9 and sg745T) and digested ("Cas/sg745T": incubated with Cas9 and sg745T guide). The white arrow indicates the band from the undigested PCR product (size of 682 bp), while black arrows correspond to the digestion result (two bands with sizes of 394 and 288 bp).

FIGURE S2

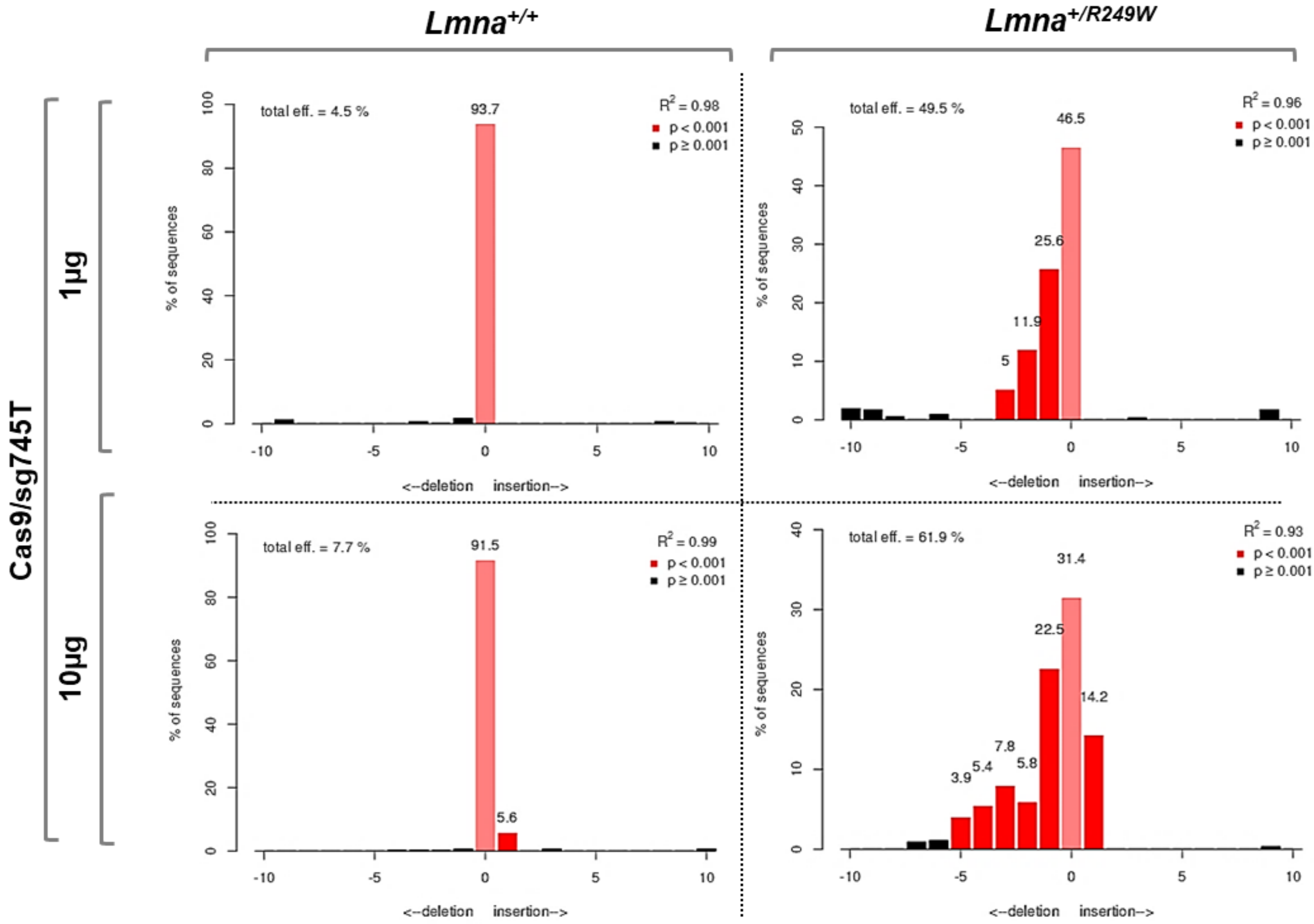

Figure S2. Indel spectrum determined by TIDE analysis in *Lmna*$^{+/+}$ and *Lmna*$^{+/R249W}$ MEFs after electroporation with Cas9/sg745T complexes. Representative data (from three biological replicates) are shown for low (1µg) and high (10µg) Cas9/sg745T treatments. Insertion and deletion frequencies are plotted as a function of indel size (bp).

FIGURE S3

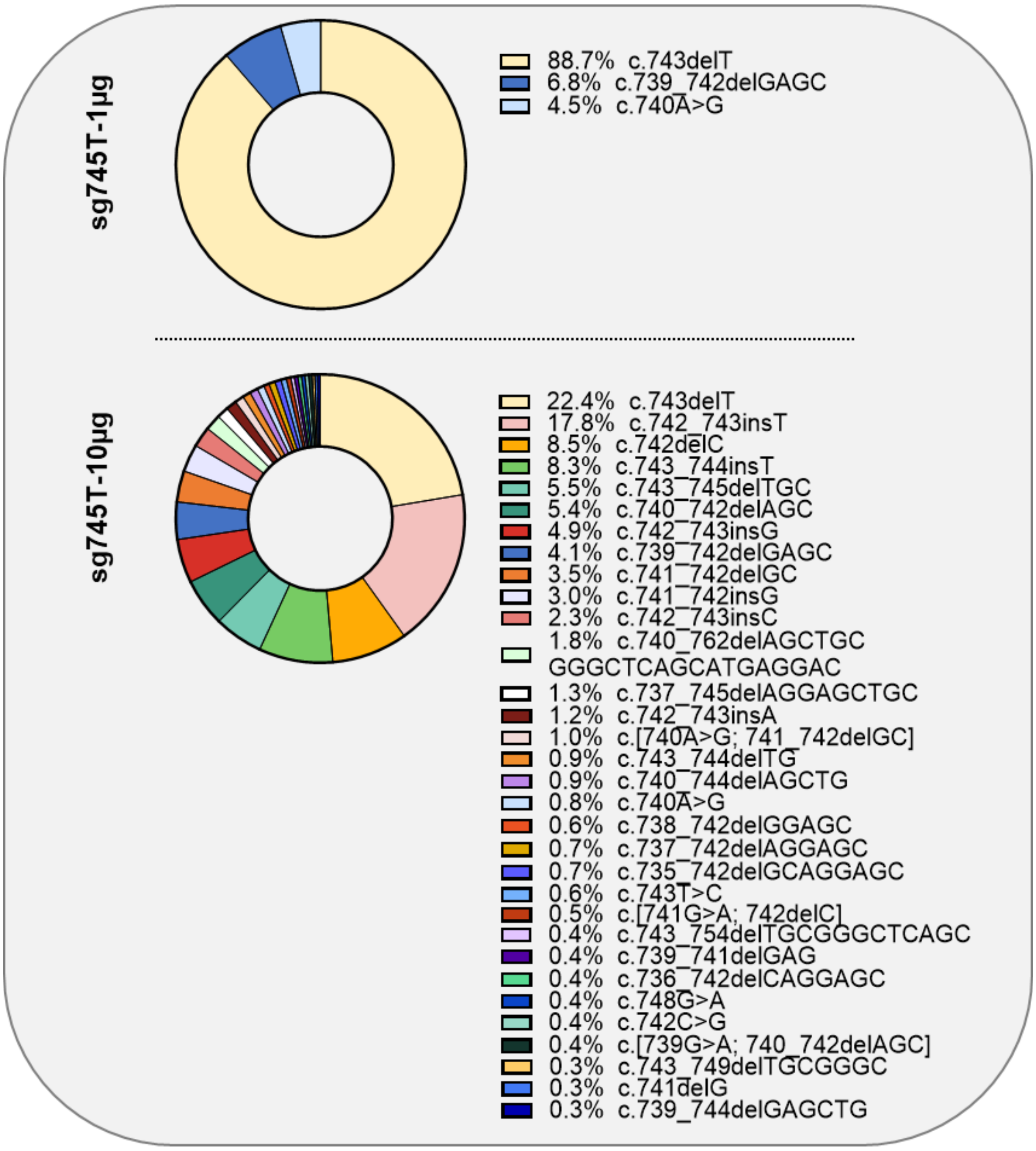

Figure S3. Distribution and frequency of CRISPR-induced indels in wild-type alleles from *Lmna*$^{+/+}$ MEFs following nucleofection with low (1µg) and high (10µg) doses of Cas9/sg745T complexes. Data represent the mean percentage of each indel type, relative to the total detected, from three experimental replicates.

FIGURE S4

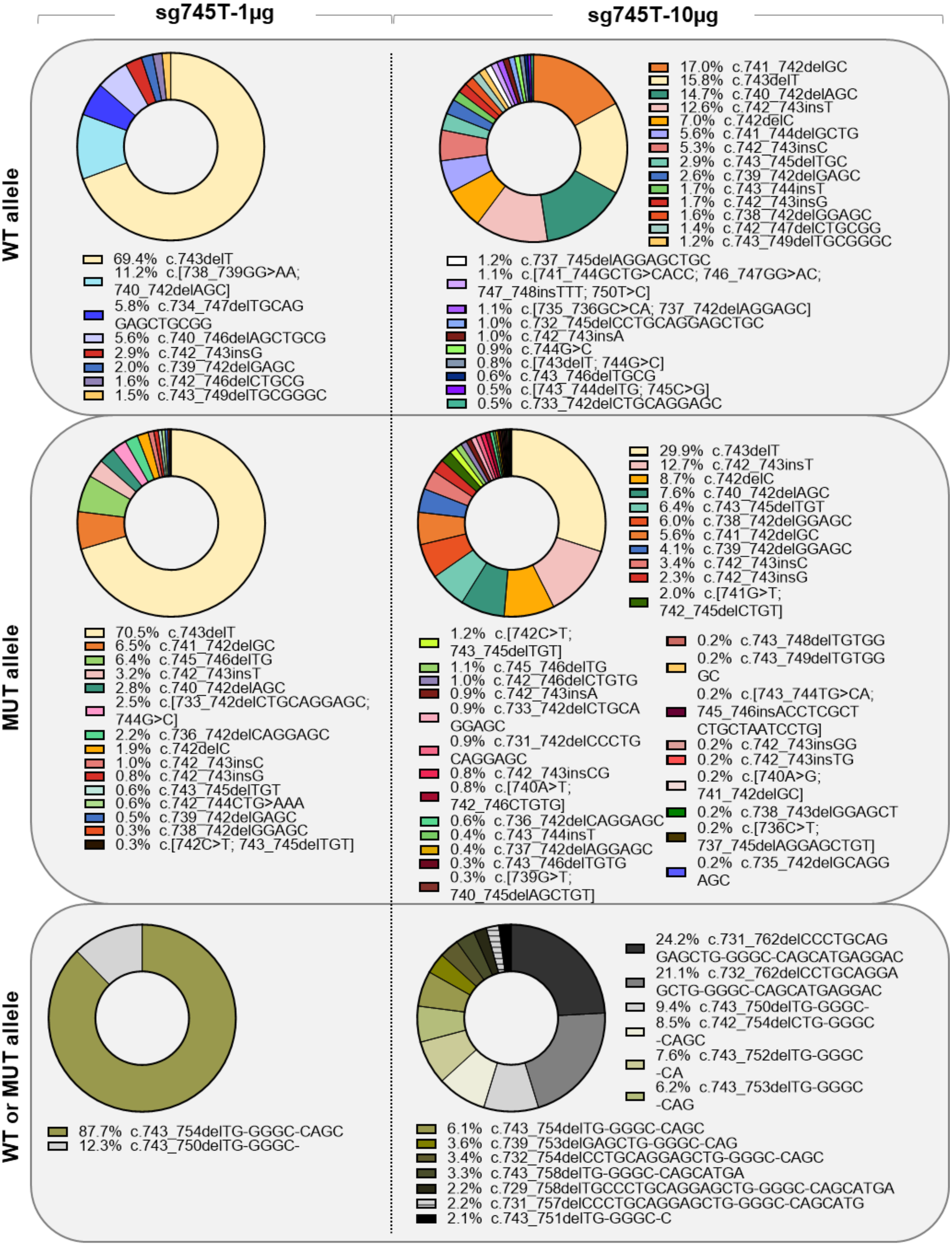

Figure S4. Distribution and frequency of CRISPR-induced indels in wild-type (WT) and mutant (MUT) alleles from *Lmna*$^{+/R249W}$ MEFs following nucleofection with low (1µg) and high (10µg) doses of Cas9/sg745T complexes. WT or MUT allele: cases where the origin of the indel-WT or MUT allele-could not be determined. Data represent the mean percentage of each indel type, relative to the total detected, from three experimental replicates.

FIGURE S5

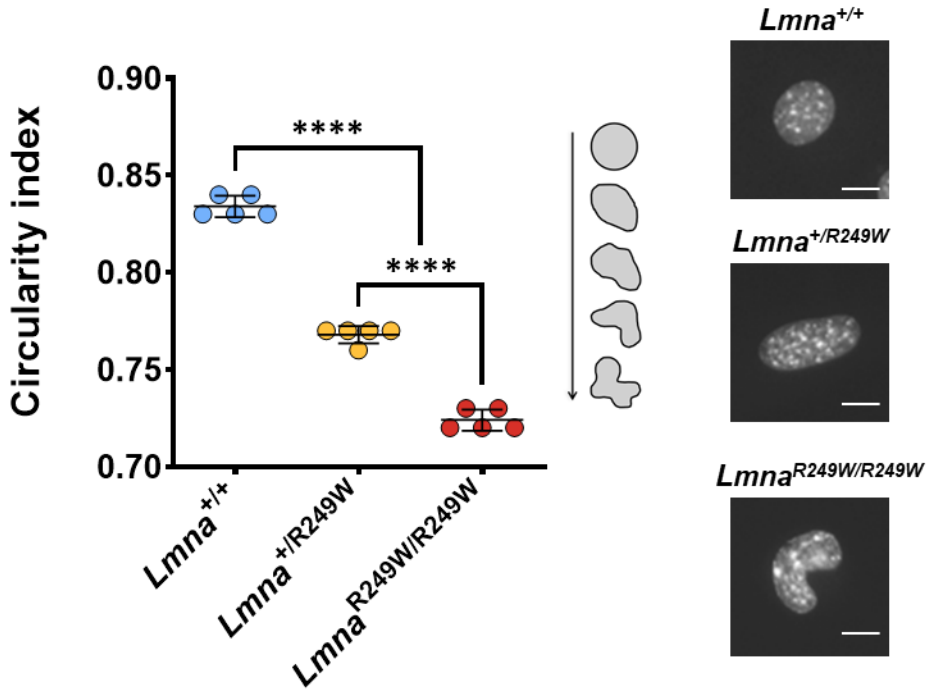

Figure S5. The nuclear morphology of MEFs containing the *Lmna* c.745C>T mutation is significantly altered. Circularity index of cell lines with three different *Lmna* genotypes (WT, heterozygous, and homozygous for the c.745C>T mutation). Data are represented as mean ± SD, with results obtained from five technical replicates (n=5). ****: P<0.0001. Representative nuclei are shown in right panels. Scale bar: 10 µm.

FIGURE S6

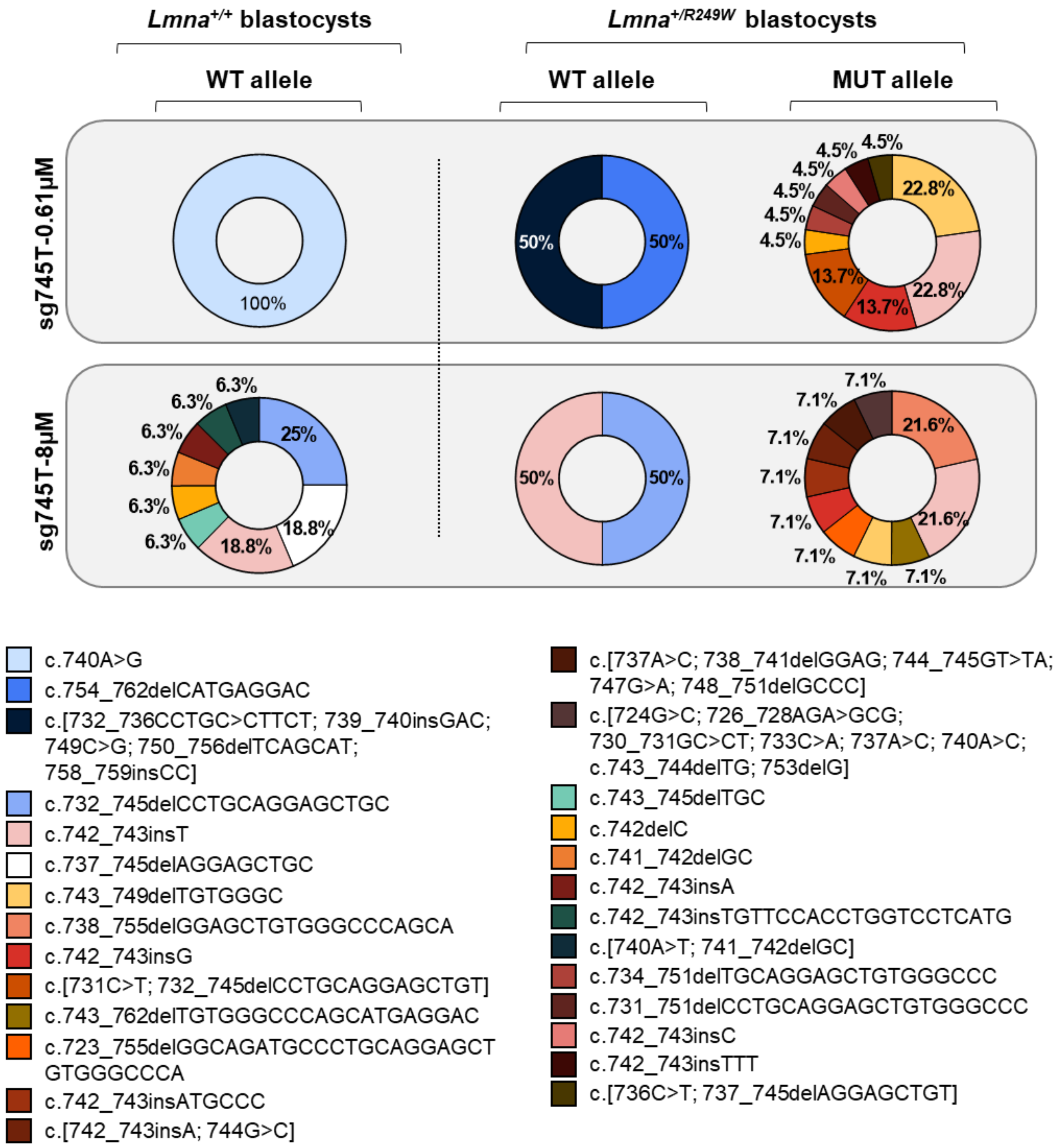

Figure S6. Frequency of blastocysts carrying CRISPR-induced indels in wild-type (WT) and mutant (MUT) alleles after nucleofection with low (0.61µM) and high (8µM) dosis of Cas9/sg745T complexes.

FIGURE S7

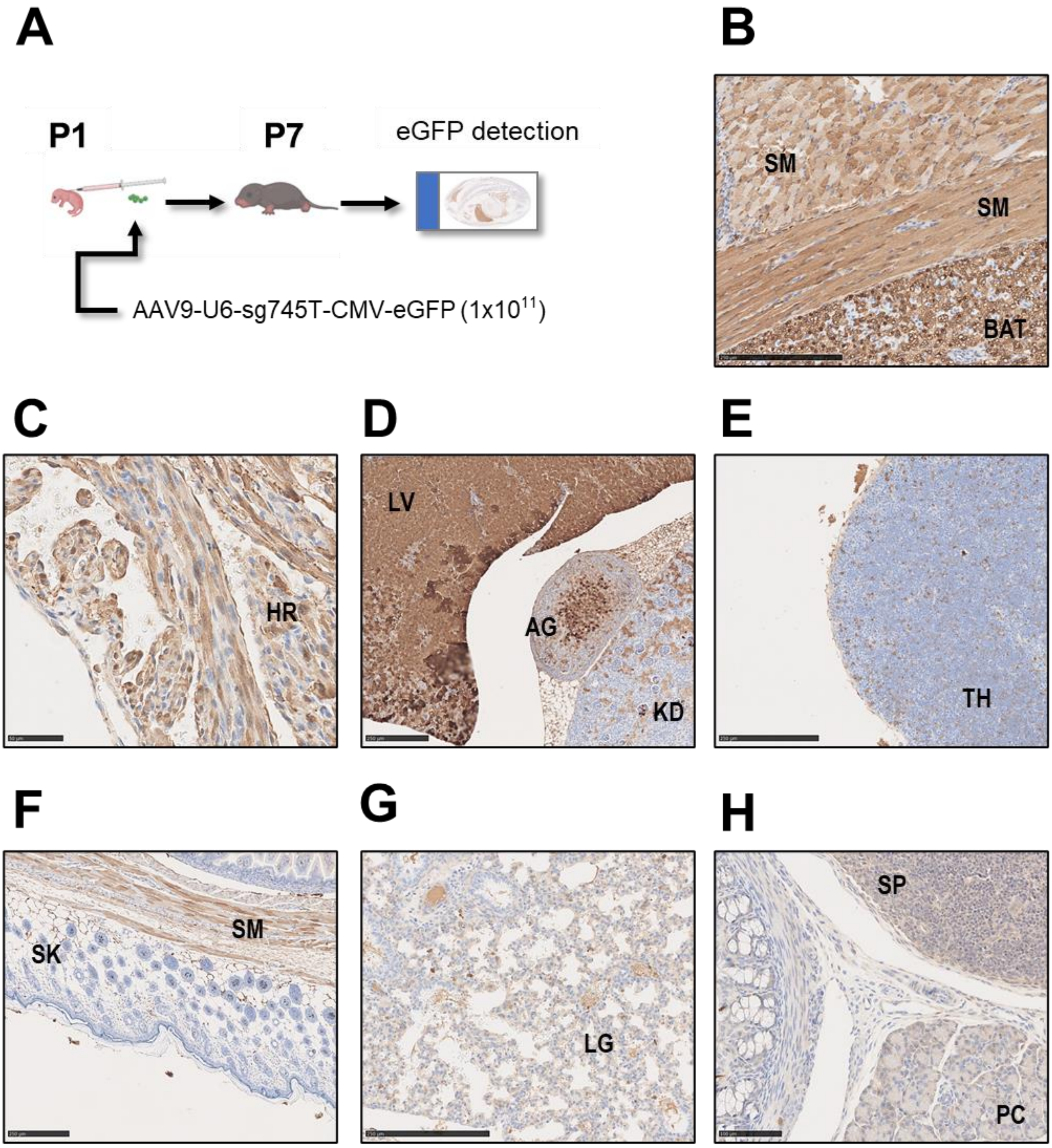

Figure S7. eGFP expression in tissues from mice infected with AAV9-U6-sg745T-CMV-eGFP. A, Newborn, wild type mice were subdermally injected at P1 with $1x10^{11}$ viral genomes of AAV9-U6-sg745T-CMV-eGFP. At P7, animals were sacrificed and tissues collected for eGFP detection by immunohistochemistry. **B-H,** Representative images showing eGFP expression in skeletal (SM), brown adipose tissue (BAT), heart (HR), liver (LV), adrenal gland (AG), kidney (KD), thymus (TH), skin (SK), lung (LG), spleen (SP) and pancreas (PC). Scale bar: 50 µm (**C**); 100 µm (**H**), 250 µm (**D-G**).

FIGURE S8

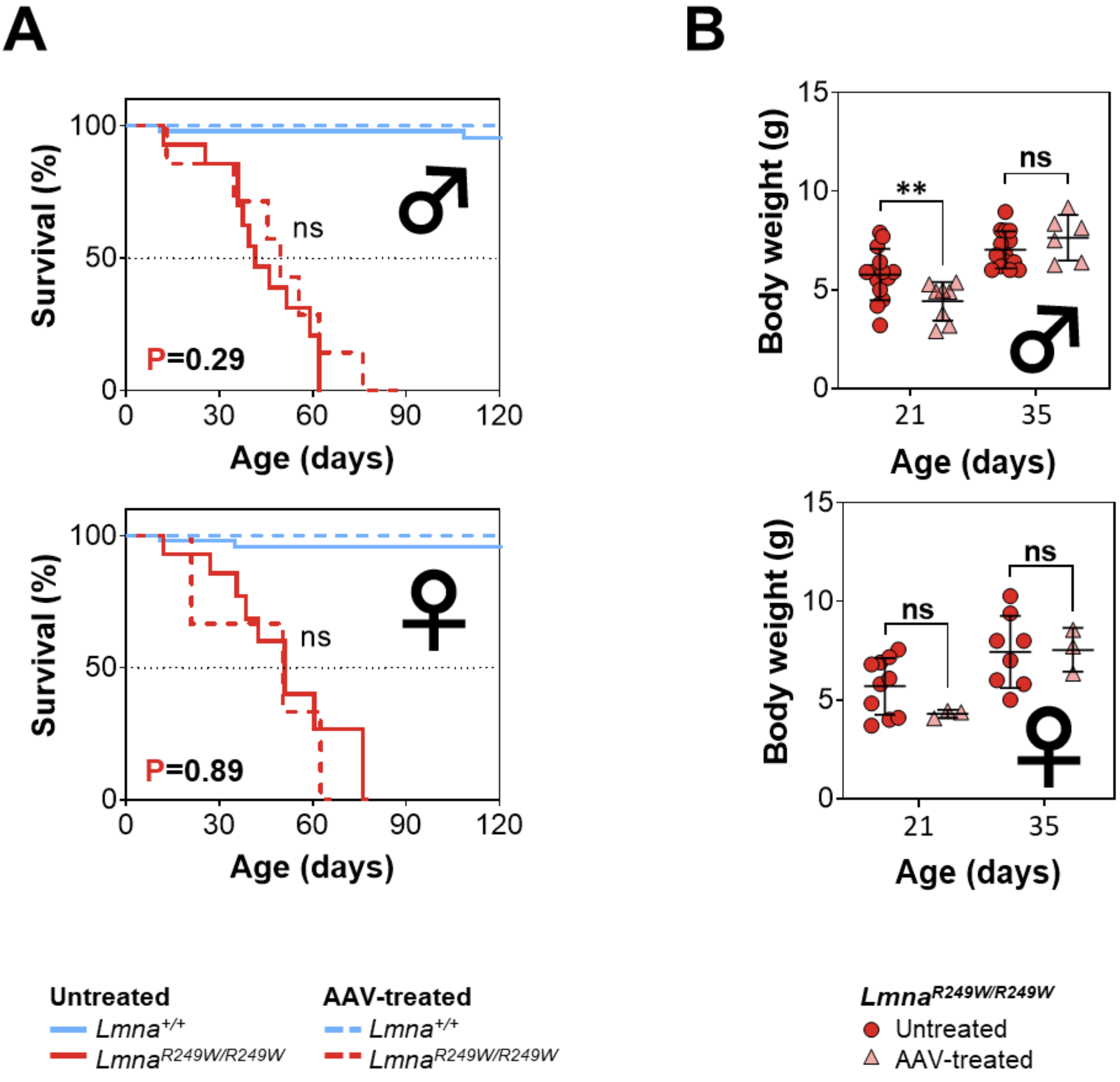

Fgure S8. Administration of AAV9-Cas9/sg745T gene therapy does not improve survival or body weight in both *Lmna*$^{R249W/R249W}$ males and females. A, Kaplan-Meier survival curve of males (upper graph) (untreated: n=50 for *Lmna*$^{+/+}$ and n=14 for *Lmna*$^{R249W/R249W}$; and AAV-treated: n=13 for *Lmna*$^{+/+}$ and n=11 for *Lmna*$^{R249W/R249W}$) and females (bottom graph) (untreated: n=50 for *Lmna*$^{+/+}$ and n=8 for *Lmna*$^{R249W/R249W}$; and AAV-treated: n=13 for *Lmna*$^{+/+}$ and n=3 for *Lmna*$^{R249W/R249W}$). **B,** Comparison of body weight in untreated and AAV-treated *Lmna*$^{R249W/R249W}$ males (upper graph) and females (bottom graph) at 21 and 35 days of age. Data are presented as mean values ± SD. ns: non-significant differences, **: P<0.01.

FIGURE S9

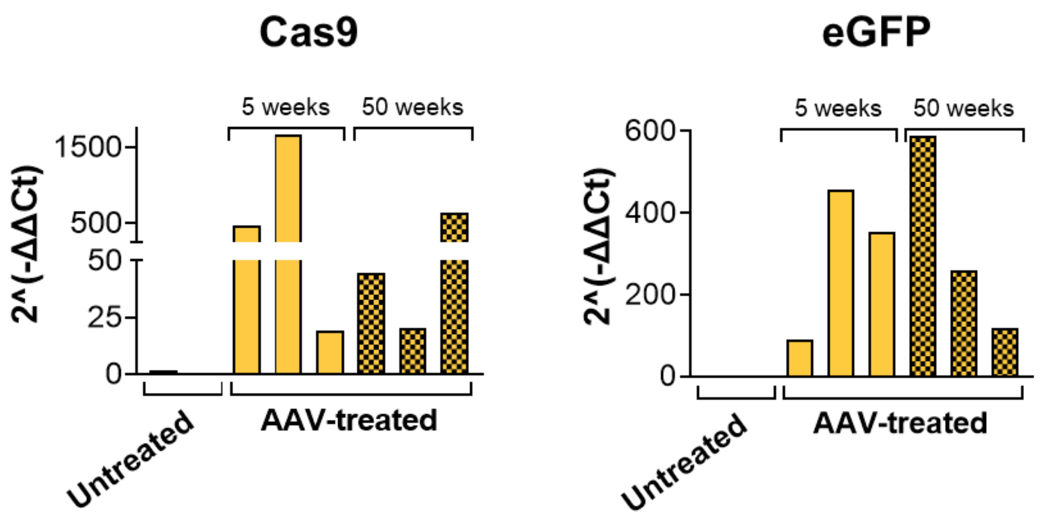

Figure S9. Cardiac expression of Cas9 and eGFP following AAV-mediated delivery in *Lmna*$^{+/R249W}$ mice. Relative mRNA levels of *Cas9* and e*Gfp* were quantified by RT-qPCR in heart tissue from AAV-treated (n=3) and untreated (n=2) mice. Expression levels were normalized to *Gapdh* mRNA levels in untreated mice.

FIGURE S10

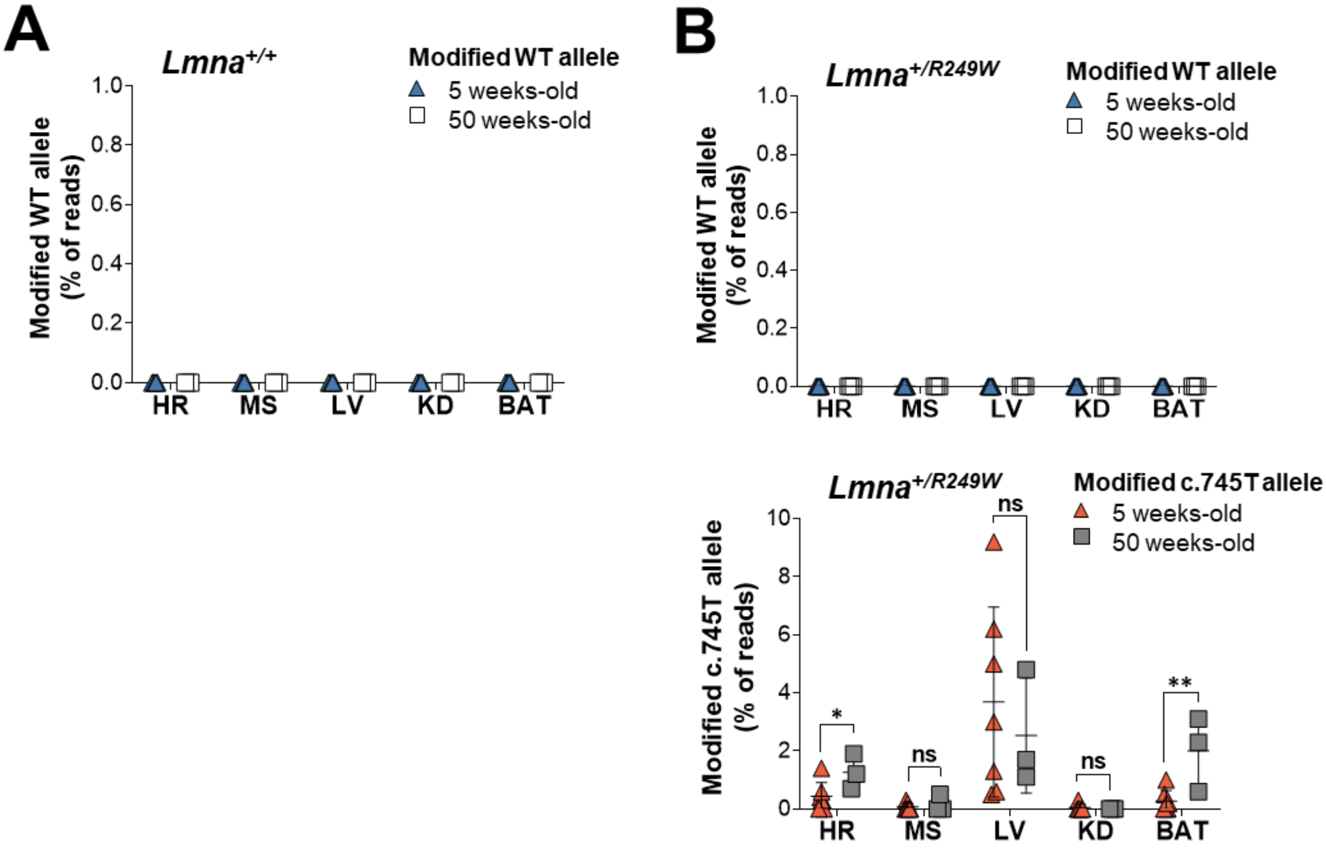

Figure S10. Quantification of indel generation in *Lmna*$^{+/+}$ and *Lmna*$^{+/R249W}$ mice infected with AAV9-Cas9/sg745T. A, Percentage of modified WT allele reads in *Lmna*$^{+/+}$ AAV-treated mice at 5 (n=5) and 50 (n=3) weeks-old. **B,** Percentage of modified WT (upper graph) and modified c.745T (bottom graph) alleles reads in *Lmna*$^{+/R249W}$ AAV-treated mice at 5 (n=7) and 50 (n=3) weeks of age. The activity of the Cas9/sg745T complex was analyzed in different tissues: heart (HR), muscle (MS), liver (LV), kidney (KD) and brown adipose tissue (BAT). Data are presented as mean values ± SD. ns: non-significant differences; *: P<0.05; **: P<0.01.

FIGURE S11

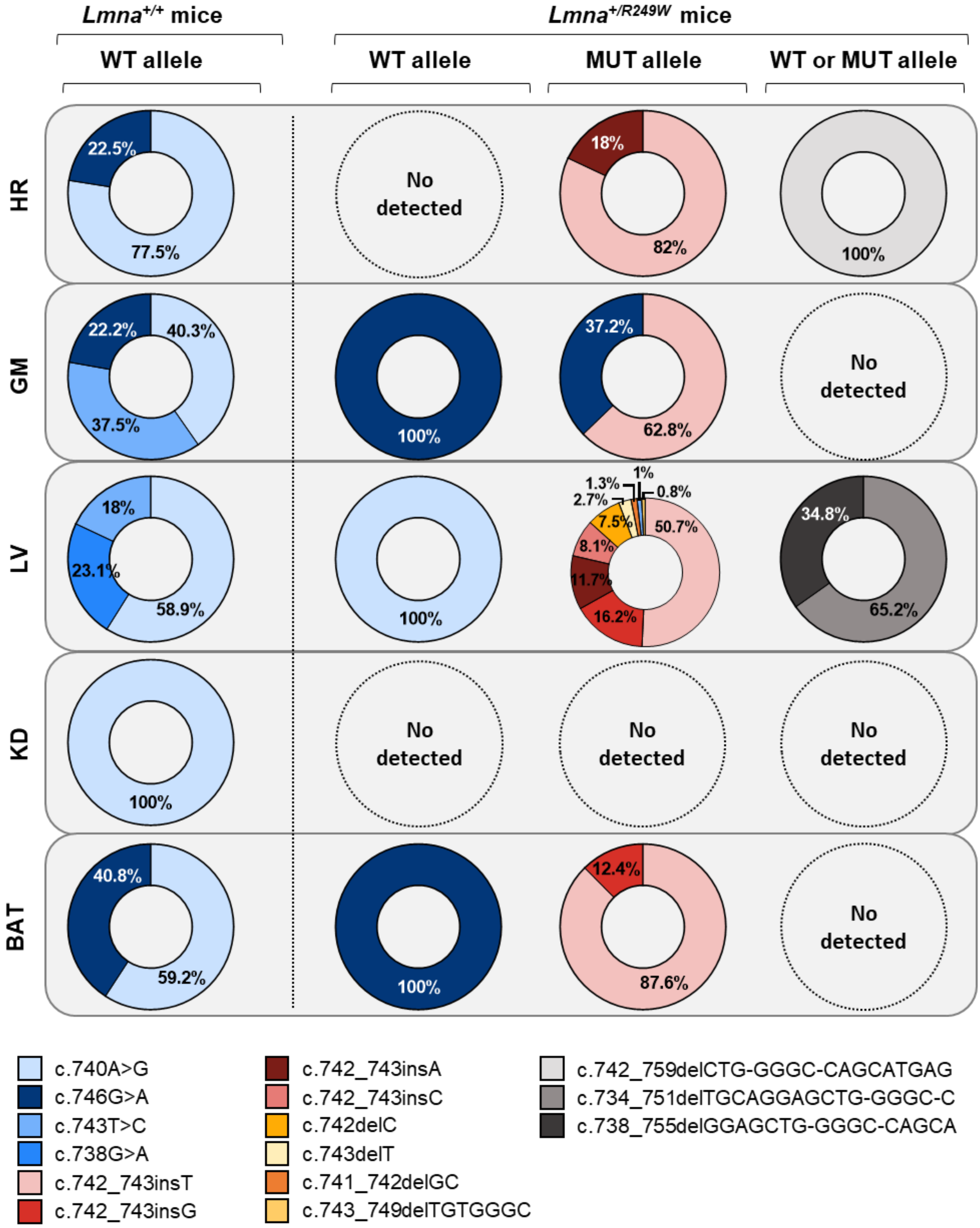

Figure S11. Distribution and frequency of CRISPR-induced indels in wild-type (WT) and mutant (MUT) alleles from both AAV-treated *Lmna*$^{+/+}$ and *Lmna*$^{+/R249W}$ mice sacrificed at 5 weeks of age. The types and frequencies of indels generated following AAV-Cas9/sg745T treatment were analyzed in heart (HR), gastrocnemius muscle (GM), liver (LV), kidney (KD) and brown adipose tissue (BAT) from WT mice (WT allele only) and heterozygous mice (WT allele, mutant allele and indels of undetermined origin-WT or MUT). Data represent the mean percentage of each indel type from four *Lmna*$^{+/+}$ and seven *Lmna*$^{+/R249W}$ mice.

FIGURE S12

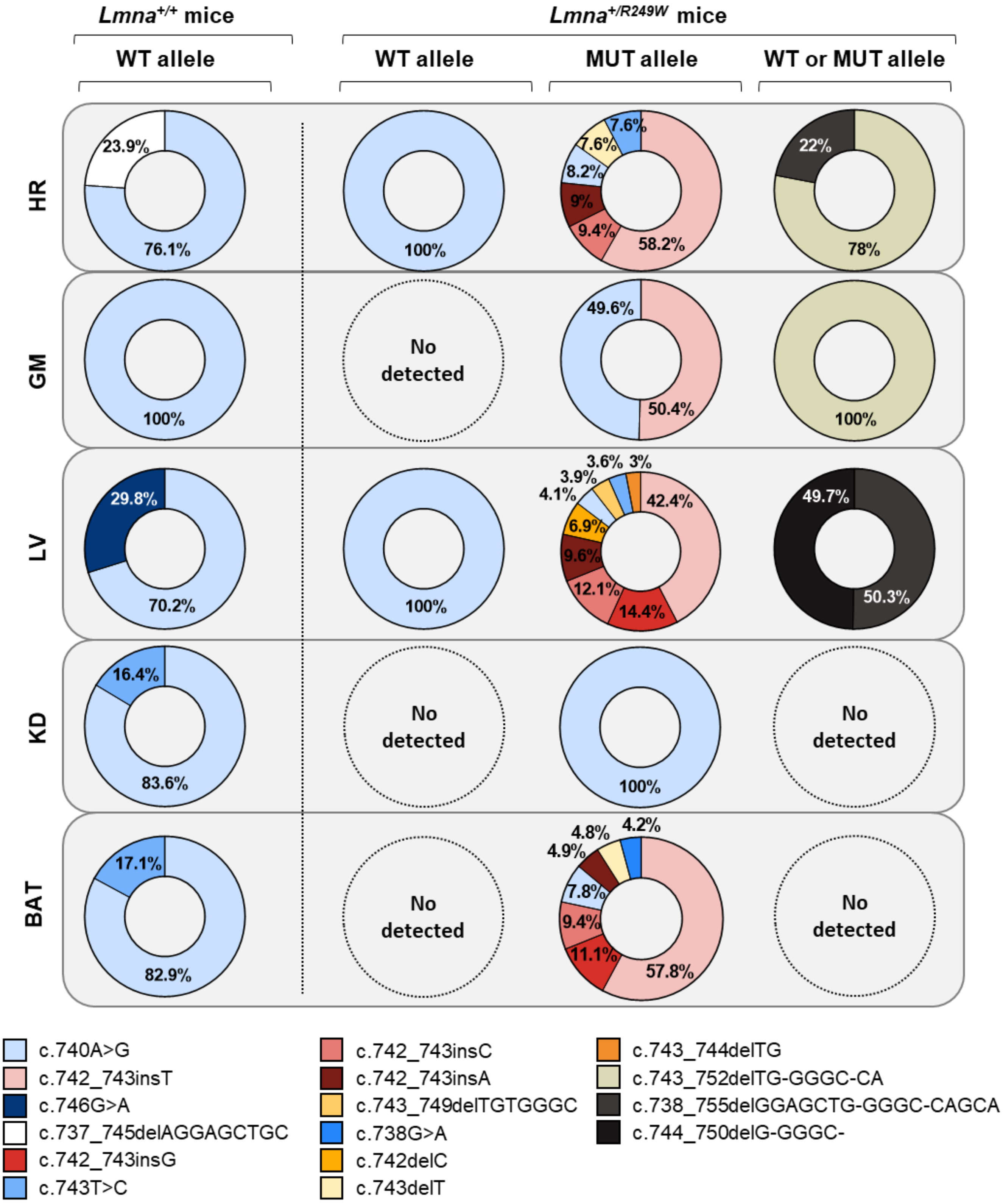

Figure S12. Distribution and frequency of CRISPR-induced indels in wild-type (WT) and mutant (MUT) alleles from both AAV-treated *Lmna*$^{+/+}$ and *Lmna*$^{+/R249W}$ mice sacrificed at 50 weeks of age. The types and frequencies of indels generated following AAV-Cas9/sg745T treatment were analyzed in heart (HR), gastrocnemius muscle (GM), liver (LV), kidney (KD) and brown adipose tissue (BAT) from WT mice (WT allele only) and heterozygous mice (WT allele, mutant allele and indels of undetermined origin-WT or MUT). Data represent the mean percentage of each indel type from four *Lmna*$^{+/+}$ and seven *Lmna*$^{+/R249W}$ mice.

FIGURE S13

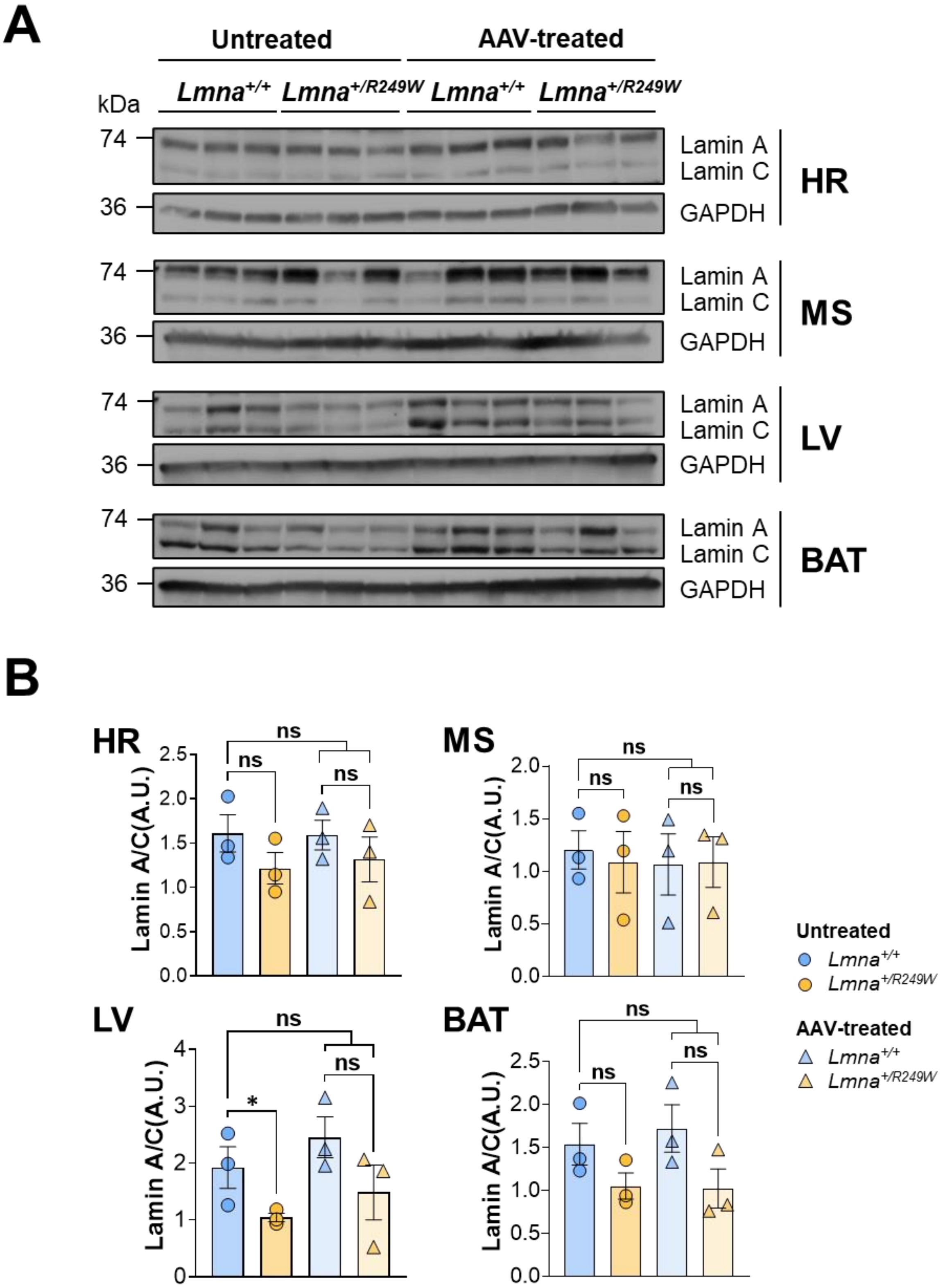

Figure S13. ***Lmna****$^{+/R249W}$* **mice treated with AAV9-Cas9/sg745T show no differences in lamin A/C expression levels compared to untreated ones. A,** Representative Western blot of lamin A/C protein expression from lysates obtained from heart (HR), muscle (MS), liver (LV) and brown adipose tissue (BAT). GAPDH was used as a loading control. **B,** Relative quantification of total lamin A/C normalized to GAPDH in heart, muscle, liver, and brown adipose tissue. Data are presented mean values ± SD. ns: non-significant differences, *: P<0.05. Samples from 3 mice aged 50 weeks were used for each of the compared groups.

FIGURE S14

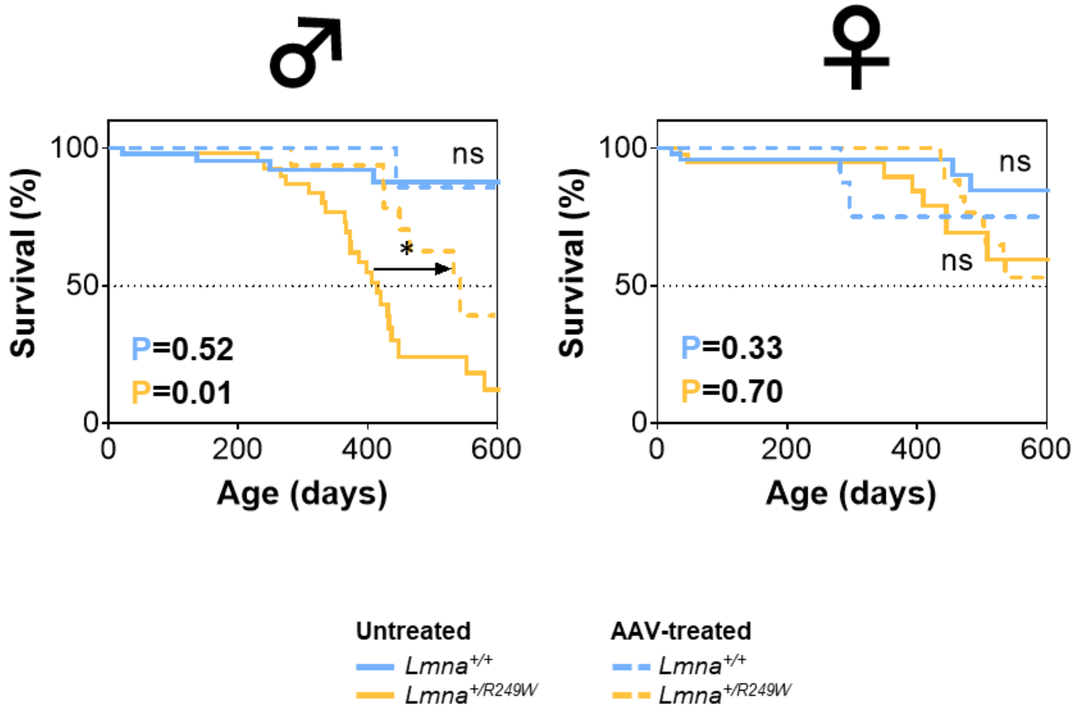

Figure S14. Administration of AAV9-Cas9/sg745T gene therapy increases survival in *Lmna*$^{+/R249W}$ males. Kaplan-Meier survival curve of males (untreated: n=50 for *Lmna*$^{+/+}$ and n=55 for *Lmna*$^{+/R249W}$; and AAV-treated: n=13 for *Lmna*$^{+/+}$ and n=16 for *Lmna*$^{+/R249W}$) and females (untreated: n=50 for *Lmna*$^{+/+}$ and n=45 for *Lmna*$^{+/R249W}$; and AAV-treated: n=13 for *Lmna*$^{+/+}$ and n=20 for *Lmna*$^{+/R249W}$).

Table S1. Predicted protein changes translated from mutant and wild type alleles containing indels (present at >5% frequency) from *Lmna*$^{+/+}$ and *Lmna*$^{+/R249W}$ MEFs after electroporation with low and high doses of Cas9/sg745T complexes.

	Protein (expected)[2]		
**CDS[1]**	**MUT protein**	**WT protein**	**Comments[3]**
**c.743delT**	p.(263*)		Mf in WT alleles of *Lmna*$^{+/+}$ MEFs of ld and hd, in WT alleles of *Lmna*$^{+/R249W}$ MEFs of ld and in mutant alleles of *Lmna*$^{+/R249W}$ MEFs of ld and hd
**c.741_742delGC**	p.(252*)		Mf in WT alleles of *Lmna*$^{+/R249W}$ MEFs of ld
c.742_743insT	p.(253*)		
c.740_742delAGC	p.(665*)		
c.[738_739GG>AA; 740_742delAGC]	ND	p.(665*)	
c.742delC	p.(263*)		
c.743_744insT	p.(253*)		
c.739_742delGAGC	p.(262*)		
c.745_746delTG	p.(252*)	ND	
c.738_742delGGAGC	p.(251*)		
c.734_747delTGCAGGAGCT GCGG		p.(248*)	
c.740_746delAGCTGCG	ND	p.(261*)	
c.741_744delGCTG		p.(262*)	
c.743_745delTGC		p.(665*)	
c.743_745delTGT	p.(665*)	ND	
c.742_743insC	p.(253*)	p.(253*)	
c.743_754delTG-GGGC-CAGC	p.(662*)		
c.731_762delCCCTGCAGGA GCTG-GGGC-CAGCATGAGGAC	p.(249*)		
c.732_762delCCTGCAGGAG CTG-GGGC-CAGCATGAGGAC	p.(253*)		
c.743_750delTG-GGGC-	p.(250*)		
c.743_752delTG-GGGC-CA	p.(260*)		
c.743_753delTG-GGGC-CAG	p.(249*)		

[1] Obtained by deep sequencing.
[2] ND (No detected): this indel is not detected in the mutant allele (if it appears in the "MUT protein" column) or in the WT allele (if it appears in the "WT protein" column).
[3] Most frequent: Mf. Low dosis: ld. High dosis: hd.

Table S2. Predicted protein changes translated from mutant and wild type alleles containing indels from *Lmna*$^{+/+}$ and *Lmna*$^{+/R249W}$ blastocysts transfected with Cas9/sg745T complexes at low and high concentrations.

CDS(1)	Protein (expected)(2) MUT protein	Protein (expected)(2) WT protein	Comments(3)
**c.740A>G**	ND	p.E247G	Mf in WT alleles of *Lmna*$^{+/+}$ blastocysts (ld)
**c.754_762delCATGAGGAC**	ND	p.(663*)	Mf in WT alleles of *Lmna*$^{+/R249W}$ blastocysts (ld)
**c.[732_736CCTGC>CTTCT; 739_740insGAC; 749C>G; 750_756delTCAGCAT; 758_759insCC]**	ND	p.(261*)	Mf in WT alleles of *Lmna*$^{+/R249W}$ blastocysts (ld)
**c.732_745delCCTGCAGGAGCTGC**	ND	p.(248*)	Mf in WT alleles of *Lmna*$^{+/+}$ blastocysts (hd) dosis
**c.742_743insT**	p.(253*)	p.(253*)	Mf in WT alleles of *Lmna*$^{+/R249W}$ blastocysts (hd) and in mutant alleles of *Lmna*$^{+/R249W}$ blastocysts (ld and hd)
**c.737_745delAGGAGCTGC**	ND	p.(663*)	Mf in WT alleles of *Lmna*$^{+/R249W}$ blastocysts (hd)
**c.743_749delTGCGGGC**	p.(261*)	ND	Mf in mutant alleles of *Lmna*$^{+/R249W}$ blastocysts (ld)
**c.738_755delGGAGCTGTGGGCCCAGCA**	p.(660*)		Mf in mutant alleles of *Lmna*$^{+/R249W}$ blastocysts (hd)
c.742_743insG	p.(253*)		
c.[731C>T; 732_745delCCTGCAGGAGCTGT]	p.(248*)		
c.743_762delTGTGGGCCCAGCATGAGGAC	p.(253*)		
c.723_755delGGCAGATGCCCTGCAGGAGC TGTGGGCCCA	p.(654*)		
c.742_743insATGCCC	p.(668*)		
c.[742_743insA; 744G>C]	p.(253*)		
c.[737A>C; 738_741delGGAG; 744_745GT>TA; 747G>A; 748_751delGCCC]	p.(250*)		
c.724G>C; 726_728AGA>GCG; 730_731GC>CT; 733C>A; 737A>C; 740A>C; 743_744delTG; 753delG]	p.(665*)		
c.743_745delTGC	ND	p.(665*)	
c.742delC	p.(263*)	p.(263*)	
c.741_742delGC	ND	p.(252*)	
c.742_743insA		p.(253*)	
c.742_743insTGTTCCACCTGGTCCTCATG		p.(270*)	

	c.[740A>T; 741_742delGC]		p.(252*)
	c.734_751delTGCAGGAGCTGTGGGCC C	p.(660*)	ND
	c.731_751delCCTGCAGGAGCTGTGGG CCC	p.(659*)	
	c.742_743insC	p.(253*)	p.(253*)
	c.742_743insTTT	p.(667*)	ND
	c.[736C>T; 737_745delAGGAGCTGT]	p.(663*)	

(1) Obtained by deep sequencing.
(2) ND (No detected): this indel is not detected in the mutant allele (if it appears in the “MUT protein” column) or in the WT allele (if it appears in the “WT protein” column).
(3) Most frequent: Mf. Low dosis: ld. High dosis: hd.

Table S3. Prediction[(1)] of the proteins translated from mutant and wild type alleles with indels obtained from both AAV-treated *Lmna*$^{+/+}$ and *Lmna*$^{+/R249W}$ mice sacrificed at 5 and 50 weeks of age.

<table>
<tr><th></th><th colspan="2">5 weeks old</th><th colspan="2">50 weeks old</th><th></th></tr>
<tr><th>Indel[(2)]</th><th>MUT allele</th><th>WT allele</th><th>MUT allele</th><th>WT allele</th><th>Comments[(3)]</th></tr>
<tr><td>c.740A>G</td><td>ND</td><td>p.E247G</td><td colspan="2">p.E247G</td><td>Mf in WT alleles of 50-week-old, Lmna$^{+/+}$ and Lmna$^{+/R249W}$ mice and in in WT alleles of 5-week-old, Lmna$^{+/+}$ mice</td></tr>
<tr><td>c.746G>A</td><td>p.(249*)</td><td>p.R249Q</td><td>ND</td><td>p.R249Q</td><td>Mf in WT alleles of 5-week-old, Lmna$^{+/R249W}$ mice</td></tr>
<tr><td>c.742_743insT</td><td>p.(253*)</td><td>ND</td><td>p.(253*)</td><td>ND</td><td>Mf in MUT alleles of 5 and 50 week-old Lmna$^{+/R249W}$ mice</td></tr>
<tr><td>c.743T>C</td><td colspan="4">p.L248P</td><td rowspan="13">-</td></tr>
<tr><td>c.738G>A</td><td>ND</td><td>NC</td><td colspan="2">NC</td></tr>
<tr><td>c.742_743insA</td><td rowspan="3">p.(253*)</td><td rowspan="3">ND</td><td rowspan="3">p.(253*)</td><td rowspan="3">ND</td></tr>
<tr><td>c.742_743insG</td></tr>
<tr><td>c.742_743insC</td></tr>
<tr><td>c.742delC</td><td rowspan="2">p.(263*)</td><td rowspan="2">ND</td><td rowspan="2">p.(263*)</td><td rowspan="2">ND</td></tr>
<tr><td>c.743delT</td></tr>
<tr><td>c.743_749delTGTGGGC</td><td colspan="2" rowspan="2">ND</td><td>p.(261*)</td><td>ND</td></tr>
<tr><td>c.743_744delTG</td><td>p.(252*)</td><td>ND</td></tr>
<tr><td>c.741_742delGC</td><td>p.(252*)</td><td>ND</td><td colspan="2" rowspan="2">ND</td></tr>
<tr><td>c.743_749delTGTGGGC</td><td>p.(261*)</td><td>ND</td></tr>
<tr><td>c.743_752delTG-GGGC-CA</td><td colspan="2" rowspan="3">ND</td><td colspan="2">p.(260*)</td></tr>
<tr><td>c.744_750delG-GGGC-</td><td colspan="2">p.(261*)</td></tr>
<tr><td>c.737_745delAGGAGCTGC</td><td>ND</td><td>p.(663*)</td><td>Detected in heart of 1/3 WT mice (0.3% of the total reads of that animal)</td></tr>
<tr><td>c.742_759delCTG-GGGC-CAGCATGAG</td><td colspan="2" rowspan="2">p.(660*)</td><td colspan="2" rowspan="2">ND</td><td>Detected in heart of 1/7 Lmna$^{+/R249W}$ mice (0.3% of the total reads of that animal)</td></tr>
<tr><td>c.734_751delTGCAGGAGCTG-GGGC-C</td><td>Detected in liver of 2/7 Lmna$^{+/R249W}$ mice (0.3% of the total reads of those animals)</td></tr>
<tr><td>c.738_755delGGAGCTG-GGGC-CAGCA</td><td colspan="4">p.(660*)</td><td>Detected in liver of 1/7 Lmna$^{+/R249W}$ mice (0.3% of the total reads in that animal)</td></tr>
</table>

(1) NC (No change): the modification does not cause any change in the protein. ND (No detected): this indel is not detected in the mutant allele (if it appears in the “MUT protein” column) or in the WT allele (if it appears in the “WT protein” column).
(2) Obtained by deep sequencing
(3) Most frequent: Mf.

Table S4. Histopathological evaluation of multiple organs from AAV-treated and untreated *Lmna*$^{+/R249W}$ mice. Three males per genotype were analyzed, all sacrificed between 46 and 51 weeks of age. Abbreviations: GM, gastrocnemius muscle; LV, liver; KD, kidney; PC, pancreas; SP, spleen; LG, lung; DPG, diaphragm; BAT, brown adipose tissue; WAT, white adipose tissue. NA: no abnormalities detected. NT: tissue not collected.

	**AAV-treated *Lmna*$^{+/R249W}$**			**Untreated *Lmna*$^{+/R249W}$**		
**Mouse ID (Age, weeks) / Tissue**	**E38(51)**	**E39(51)**	**E61(51)**	**R249_85(42)**	**R249_173(49)**	**R249_174(46)**
**GM**	**NA**	**NA**	**NA**	**NA**	**NA**	**NA**
**LV**	**NA** Glyclogen (++)	**NA** Glycogen (++)	**NA** Glycogen (++)	**NA** Glycogen (+++)	Anisocaryosis and low glycogen (+)	Anisocaryosis and low glycogen (+)
**KD**	Tubular epithelial vacuolization +/Perivascular and peripelvic mononuclear inflammatory infiltration ++/Tubular basophilia +	Tubular epitelial vacuolization ++/Perivascular and peripelvic mononuclear inflammatory infiltration ++	Tubular epitelial vacuolization ++/Perivascular and peripelvic mononuclear inflammatory infiltration ++	Perivascular and peripelvic mononuclear inflammatory infiltration +	Perivascular and peripelvic mononuclear inflammatory infiltration +	Severe tubular epithelial vacuolization +++/ Perivascular and peripelvic mononuclear inflammatory infiltration +
**PC**	**NA**	**NA**	**NA**	**NA**	**NA**	**NA**
**SP**	**NA**	**NA**	**NA**	**NA**	**NA**	**NA**
**LG**	**NA** Small foci of perivascular / peribronchial + mononuclear infiltration	Foci of perivascular mononuclear infiltration / very prominent peribronchial +++/ Collapsed and congestive	Acidophilic macrophage pneumonia / Histiocitic sarcoma / Edema	NT	Foci of perivascular mononuclear infiltration / Peribronchial + / Foci of non-suppurative pneumonia / Collapsed and congestive	Foci of perivascular mononuclear infiltration / very prominent peribronchial +++ / Foci of non-suppurative pneumonia / Collapsed and congestive /Bronchoalveolar adenoma
**DPG**	**NA** Lymphoid accumulations (diaphragmatic nodule)	**NA** Lymphoid accumulations (diaphragmatic nodule)	Lymphoid accumulations (diaphragmatic nodule); minimal degenerative focus	**NA** Lymphoid accumulations (diaphragmatic nodule)	**NA**	**NA** Lymphoid accumulations (diaphragmatic nodule)
**BAT**	Lipodystrophy: sparse multilocular vacuole (++)	Lipodystrophy: sparse multilocular vacuole (+++)	Lipodystrophy: sparse multilocular vacuole (+++)	**NA** Abundant multilocular vacuole	**NA** Abundant multilocular vacuole	**NA** Abundant multilocular vacuole
**WAT**	Lipodystrophy: Small vacuole (+++) Crushed	Lipodystrophy: Small vacuole (++) Crushed	Lipodystrophy: Small vacuole (+++) Crushed	**NA** Large unilocular vacuole	**NA** Large unilocular vacuole	**NA** Large unilocular vacuole

Table S5. Most frequent indels found in *Lmna*^{R249W} MEFs, embryos and tissues treated with Cas9/sgRNA complexes.

Targeted Allele [2]	c.740A>G p.E247G	c.746G>A p.R249Q	c.742_743 insT p.(253*)	c.743 delT p.(263*)	c.741_742 delGC p.(252*)	Multiple[1] p.(663*) p.(261*)	c.743_749 delTGTGG GC p.(261*)	c.732_745 delCCTGC AGGAGCT GC p.(248*)	c.738_755 delGGAGCTGT GGGCCCAGC A p.(660*)
**WT** (5-wo *Lmna*$^{+/+}$ **mice**)	√								
**WT** (5-wo *Lmna*$^{R249W/+}$ **mice**)		√							
**WT** (50-wo *Lmna*$^{+/+}$ **mice**)	√								
**WT** (50-wo *Lmna*$^{R249W/+}$ **mice**)	√								
**MUT** (5-wo *Lmna*$^{R249W/+}$ **mice**)			√						
**MUT** (50-wo *Lmna*$^{R249W/+}$ **mice**)			√						
**WT** (*Lmna*$^{+/+}$ **MEFs** ld)				√					
**WT** (*Lmna*$^{R249W/+}$ **MEFs** ld)				√					
**WT** (*Lmna*$^{+/+}$ **MEFs** hd)				√					
**WT** (*Lmna*$^{R249W/+}$ **MEFs** hd)					√				
**MUT** (*Lmna*$^{R249W/+}$ **MEFs** ld)				√					
**MUT** (*Lmna*$^{R249W/+}$ **MEFs** hd)				√					
**WT** (*Lmna*$^{+/+}$ **embryos** ld)	√								
**WT** (*Lmna*$^{R249W/+}$ **embryos** ld)						√			
**WT** (*Lmna*$^{+/+}$ **embryos** hd)								√	
**WT** (*Lmna*$^{R249W/+}$ **embryos** hd)			√					√	
**MUT** (*Lmna*$^{R249W/+}$ **embryos** ld)			√				√		
**MUT** (*Lmna*$^{R249W/+}$ **embryos** hd)			√						√

In **WT** alleles (12)	**4/12**	1/12	-	3/12	1/12	1/12	-	2/12	-
In **MUT** alleles (6)	-	-	**4/6**	2/6	-	-	1/6	-	1/6
In **all** alleles (18)	4/18	1/18	4/18	**5/18**	1/18	1/18	1/18	2/18	-

[1] c.754_762delCATGAGGAC and c.[732_736CCTGC>CTTCT; 739_740insGAC; 749C>G; 750_756delTCAGCAT; 758_759insCC]
[2] ld: low dose; hd: high dose

Table S6. Resources used in this work.

**Antibodies (working dilution used)**
GAPDH (14C10) rabbit mAb (1:200) / Cell Signaling (Danvers, MA, USA) (#2118)
HRP-labelled anti-rabbit secondary antibody (1:5000) / GE Healthcare (Chicago, IL, USA) (NA934-1mL)
Lamin A/C rabbit polyclonal antibody (1:2000) / Proteintech (Manchester, UK) (10298-1-AP)
**Cell culture media**
DMEM (Dulbecco's modified Eagle's medium) high glucose / Invitrogen (Waltham, MA, USA) (61965-026)
Fetal bovine serum / Sigma-Aldrich (St. Louis, MI, USA) (#F7524-500mL)
KSOM medium / Sigma (St. Louis, MI, USA) (MR-101-D)
LiteOil Global® mineral oil / DiviLab (Buenos aires, Argentina) (LGOL-100, 100mL)
M2 medium / Sigma-Aldrich (St. Louis, MI, USA) (M7167-100mL)
Penicillin/streptomycin / Lonza (Basel, Switzerland) (#DE17-602E)
**Plasmids**
pX459 vector (pSpCas9(BB)-2A-Puro) / Addgene (Watertown, MA, USA) (#62988)
pX551-CMV-SpCas9 vector / Addgene (Watertown, MA, USA) (#107024)
U6-sg745T_CMV-EGFP vector / VectorBuilder (Chicago, IL, USA)
**Reagents**
Alt-R® CRISPR-Cas9 crRNA, 10 nmol / IDT (Newark, NJ, USA)
Alt-R® CRISPR-Cas9 tracrRNA, ATTO™ 550, 20 nmol / IDT (Newark, NJ, USA) (1075928)
BD Safet-GlideTM Insulin, 3/10mL 31G x 5/16 TW / Becton Dickinson (Flankin Lakes, NJ, USA) (305937)
Cell culture microplate 96 wells / Greiner Bio-one (Kremsmünster, Austria) (655986)
Chloroform / Sigma Aldrich (St. Louis, MI, USA) (LOT#STBG0507V)
Criterion TGX Stain-Free Precast Gels / Bio-Rad (Hercules, CA, USA) (#5678084)
ECL western blotting system / ThermoFisher Scientific (Waltham, MA, USA) (32106)
EDTA Disodium salt dihydrate / Panreac (Castellar del Vallès, Spain) (131669.1210)
Equine serum gonadotropin hormone / Sigma (St. Louis, MI, USA) (G4877, 9002-70-4)
Ethanol / Panreac AppliChem (Castellar del Vallès, Spain) (131086.1612)
Fast SYBRTM Green Master Mix / ThermoFisher Scientific (Waltham, MA, USA) (3203499)
Formaldehyde / VWR BDH Chemicals (Mumbai, India) (11699408)
GenCrispr Cas9 nuclease / GenScript (Piscataway, NJ, USA) (Z03393)
Hoechst 33324 / ThermoFisher Scientific (Waltham, MA, USA) (H3570)
Human chorionic gonadotropin hormone / Sigma (St. Louis, MI, USA) (CG-10, 9002-61-3)
Hyaluronidase / StemCell (Vancouver, BC, Canada) (#07461)
Indices / Nextera XT index kit / Illumina (San Diego, CA, USA)
ISOFLUTEK® 1000 mg/g / Laboratorios Karizoo (Caldes de Montbui, Spain) (100603)
Isopropanol / Honeywell (Charlotte, NC, USA) (33539-2.5L)
Methanol / Panreac AppliChem (Castellar del Vallès, Spain) (#131091.1612)
NaOH / Panreac (Castellar del Vallès, Spain) (131659)
Nuclease Free Water / IDT (Newark, NJ, USA) (10-04-02-01)
Oligo(dT)20 / ThermoFisher Scientific (Waltham, MA, USA) (18418020)
Opti-MEM™ reduced serum medium / Gibco (Waltham, MA, USA) (31985-070)
PBS (phosphate-buffered saline) / Lonza (Basel, Switzerland) (#BE17-515Q)
Puromycin / InvivoGen (San Diego, CA, USA) (ant-pr-1)
SDS / Sigma (St. Louis, MI, USA) (L3771-100G)
Superscript III First-Strand Synthesis System / ThermoFisher Scientific (Waltham, MA, USA) (18080-051)
Trans-Blot Turbo Midi 0.2 µm Nitrocellulose Transfer Packs / Bio-Rad (Hercules, CA, USA) (#1704159)
Tris / Panreac (Castellar del Vallès, Spain) (A1086,1000)
Trizol / Invitrogen (Waltham, MA, USA) (15596018)
Tween-20 / Fisher Bioreagents (Waltham, MA, USA) (BP337-100)
**Comercial kits**
E.Z.N.A.® Tissue DNA Kit (V-Spin) / Omega Bio-Tek (Norcross, GA, USA) (D03396-02)
E.Z.N.A.® Gel Extraction kit (V-Spin column) / Omega Bio-Tek (Norcross, GA, USA) (D2500-02)
**Platforms and softwares**
Breaking Cas Design (https://bioinfogp.cnb.csic.es/tools/breakingcas/)
TIDE (https://tide.nki.nl/); CRISPResso2 (http://crispresso.pinellolab.partners.org/)
ImageJ (US National Institutes of Health, Bethesda, MD, USA)
NDP.view2 (Hamamatsu)
Prism 8 (GrahpPad Sowtware, Inc, San Diego, CA, USA)
Vevo LAB (FUJIFILM VisualSonics, Inc)

Table S7. Primer and RNA guides sequences used in this work. [1]

Primer name and 5' to 3' sequence
Lmna-Ex3-Fw: CTGGGAGAGGCTAAGAAGCA
Lmna-Ex5-Rv: GTCAATGCGGATTCGAGACT
Genotyping-Fw: CTTCTGCCATGTAGGCTCTAAG
Genotyping-Rv: ATGCCAAAGGAGAGGTGATG
DeepSeq-Fw: tcgtcggcagcgtcagatgtgtataagagacagGCTTCTAAGGAACCATTGCGA
DeepSeq-Rv: gtctcgtgggctcggagatgtgtataagagacagACCAGGGAGAGGACAGGAT
Cas9-Fw: AAACAGCAGATTCGCCTGGA
Cas9-Rv: TCATCCGCTCGATGAAGCTC
eGFP-Fw: AGTCCGCCCTGAGCAAAGA
eGFP-Rv: TCCAGCAGGACCATGTGATC
GAPDH-Fw: TGTGTCCGTCGTGGATCTGA
GAPDH-Rv: CCTGCTTCACCACCTTCTTGA
**RNA guide name and 5' to 3' sequence**
sg745T: AGATGCCCTGCAGGAGCTGT
sgScramble: GTGTAGTTCGACCATTCGTG

[1] Additional sequences added to the primer sequence for Deep sequencing PCR are indicated in lower case.

Table S8. Nucleofection conditions for mouse embryonic fibroblasts using NEPA21 electroporator.

Poring pulse					
**Voltage**	**Lenght (ms)**	**Interval (ms)**	**No.**	**D. Rate (%)**	**Polarity**
200	5	50	2	10	+
**Transfer pulse**					
**Voltage**	**Lenght (ms)**	**Interval (ms)**	**No.**	**D. Rate (%)**	**Polarity**
20	50	50	5	40	+/-

Table S9. Nucleofection conditions for mouse embryos using NEPA21 electroporator.

Poring pulse					
**Voltage**	**Lenght (ms)**	**Interval (ms)**	**No.**	**D. Rate (%)**	**Polarity**
225	1.5	50	4	10	+
**Transfer pulse**					
**Voltage**	**Lenght (ms)**	**Interval (ms)**	**No.**	**D. Rate (%)**	**Polarity**
20	50	50	5	40	+/-
